# Microsatellite Variations of Elite Setaria Varieties Released during Last Six Decades in China

**DOI:** 10.1371/journal.pone.0125688

**Published:** 2015-05-01

**Authors:** Guanqing Jia, Xiaotong Liu, James C. Schnable, Zhengang Niu, Chunfang Wang, Yuhui Li, Shujun Wang, Suying Wang, Jinrong Liu, Erhu Guo, Hui Zhi, Xianmin Diao

**Affiliations:** 1 Institute of Crop Sciences, Chinese Academy of Agricultural Sciences, Beijing, P. R. China; 2 Institute of Millet Crops, Shanxi Academy of Agricultural Sciences, Changzhi, Shanxi, P. R. China; 3 Institute of Millet Crops, Anyang Academy of Agricultural Sciences, Anyang, Henan, P. R. China; National Institute of Plant Genome Research, INDIA

## Abstract

Crop improvement is a multifaceted micro-evolutionary process, involving changes in breeding approaches, planting configurations and consumption preferences of human beings. Recent research has started to identify the specific genes or genomic regions correlate to improved agronomic traits, however, an apparent blank between the genetic structure of crop elite varieties and their improving histories in diverse modern breeding programs is still in existence. Foxtail millet (*Setaria italica*) was one of the earliest cereal crops to be domesticated and served as a staple crop for early civilizations in China, where it is still widely grown today. In the present trial, a panel of foxtail millet elite varieties, which were released in the last sixty years in different geographical regions of China, was characterized using microsatellite markers (SSRs). A clear separation of two subpopulations corresponding to the two eco-geographical regions of foxtail millet production in China was identified by the dataset, which also indicated that in more recently released elite varieties, large quantities of accessions have been transferred from spring-sowing to summer-sowing ecotypes, likely as a result of breeding response to planting configurations. An association mapping study was conducted to identify loci controlling traits of major agronomic interest. Furthermore, selective sweeps involved in improvement of foxtail millet were identified as multi-diverse minor effect loci controlling different agronomic traits during the long-term improvement of elite varieties. Our results highlight the effect of transition of planting configuration and breeding preference on genetic evolvement of crop species.

## Introduction

Foxtail millet (*Setaria italica* (L.) P. Beauv.) was one of the earliest crop species to be domesticated. It has been grown as a crop in China for more than 8,700 years and recent archeological evidence has pushed the earliest evidence for the domestication of this crop even further back to 11,500 years before present [1–2]. Currently, foxtail millet is grown on approximately 2 million hectares each year in China, and produces nearly 6 million tons of grain per year [3]. In addition to its agricultural importance, interest has also been growing in the use of foxtail millet as a model species for addressing biological questions related to abiotic stress tolerance and the evolution of C_4_ photosynthesis. The advantages of using foxtail millet to address these questions include its small genome (515Mb), the lack of recent polyploidy events in its evolutionary history, high level of genetic diversity, and self pollinating nature [4–5]. Foxtail millet and its wild ancestor green foxtail (*Setaria viridis*) are currently being used to decipher the molecular basis of C_4_ photosynthesis, with the potential to create C_4_ rice with as much as a 50% yield increase [6]. These same advantages allow foxtail millet to be used as a genetic model for closely related biofuels crops such as switch grass (*Panicum virgatum*) and napiergrass (*Pennisetum purpureum*), whose large polyploid genomes render traditional genetic and genomic approaches impractical [7–8]. The species may also provide new insights into gene networks in maize, a paleotetraploid species where many key regulatory genes are present as duplicate pairs, reducing the chances of identifying these genes through forward genetics and increasing the difficulty of reverse genetics approaches [5]. The release of a draft genome sequence for the elite foxtail millet elite variety Yugu1 and the construction of a haplotype map of genome variation within foxtail millet are accelerating the development of foxtail millet and green foxtail (both species are commonly referred to as “Setaria” in the scientific literature) as a model system [4, 9–11].

While basic biology research is being conducted using Setaria around the world, efforts to develop improved elite varieties of foxtail millet are centered in China [3]. Over 40 programs/institutes across China worked on foxtail millet breeding between the 1950s and the 1970s. At that time most breeding programs were primarily focused on line selection and comparison among landraces. In the 1980s and 1990s, these methods were displaced by pedigree selection among hybrid derivatives of different varieties and selection from mutation lines generated using radiation and other mutagenesis methods. A great deal of progress was made in these decades in developing lodging-resistant and higher yielding elite varieties. Although many elite varieties with minor differences in traits were released in the late 1990s to 2010s, fewer major improvements in agronomic performance were achieved, with the notable exception of the successful transfer of herbicide resistant genes from wild green foxtail to foxtail millet [12]. Over the past six decades roughly 550 foxtail millet elite varieties were registered with local or central authorities in China. These elite varieties were developed by more than 40 separate foxtail millet breeding programs located in eleven different Chinese provinces where foxtail millet remains a traditional staple food. Two types of cropping pattern of Setaria including both spring-sowing (one year one harvest system) and summer-sowing (one year two harvest system) were utilized by farmers to efficiently improve total cereal grain yield for insurance of food security in China [3].

In order to efficiently utilize germplasm in breeding programs, it is necessary to understand the level of diversity and any population structure present in the population. While there have been many studies focused on the genetic relationships and population structure of samples conserved at the International Crops Research Institute for the Semi-Arid Tropics (ICRISAT, India) including a number of Chinese landrace accessions [13–16] to date, no published studies have focused on the genetic structure and diversity of elite Chinese foxtail millet varieties developed in the modern era. In terms of marker assisted breeding in foxtail millet, several QTLs for branching, tillering and flowering time characters [17–18] and inflorescence morphology [19] in foxtail millet have been identified through genetic linkage analysis. Genome Wide Association Studies or GWAS of agronomic traits of a world-wide panel of accessions has identified 520 genomic regions significantly correlated with morphology, yield and growth time characters [11] in foxtail millet. Nevertheless, more QTLs remain to be dissected due to the significant population structure inferred in diverse panel of foxtail millet accessions [20].

Here we describe the population structure of a set of 348 elite varieties selected to be a representative sample of the diversity found across all the eco-regions of China in which foxtail millet is domesticated [21] and cultivated and to encompass the changes in population structure from the transition between landraces [14] to elite varieties as a result of changes in breeding and cultivation practices over the past 60 years. The marker data generated was also employed to search for loci linked to variation in key agronomic traits using association mapping and to identify loci where diversity was reduced as a result of selection in the transition from landrace to elite varieties.

## Materials and Methods

### Foxtail millet elite variety sampling

A total of 348 foxtail millet elite varieties released during the last 60 years from 41 breeding programs/institutes located in eleven provinces in Northern China were used, which represent all the ecotypes found in foxtail millet growing regions [14] (**Table 1**). In South China, foxtail millet is only cultivated as a minor crop on marginal land. As a result, farmers in southern China still use landrace varieties with no registered elite varieties being released in the past six decades. Therefore no samples from South China were included in this study. All varieties mentioned in this investigation are kept in China National Germplasm Bank (CNGB). All varieties were grown in experimental station of CAAS (Chinese Academy of Agricultural Sciences) in Shunyi district of Beijing city during 2011 summer growing season. Leaves of one single individual per accessions were sampled at the stage of seedling and were stored under -80°C.

**Table 1:** List of 348 foxtail millet improved elite varieties collected from eleven provinces.

Code	Released administrative provinces	No. of breeding programs/institutes	No. of varieties
1	Beijing	4 (1)^※^	8
2	Gansu	1 (1)	11
3	Hebei	7 (6)	78
4	Heilongjiang	7 (2)	50
5	Henan	2 (2)	19
6	Jilin	5 (2)	44
7	Liaoning	3 (2)	9
8	Inner Mongolia	4 (2)	45
9	Shandong	1 (1)	19
10	Shannxi	2 (2)	11
11	Shanxi	5 (4)	54

※The numbers outside the bracket indicate the numbers of breeding programs/institutes in the 1970s and 1980s, and that inside the bracket indicate the numbers of breeding programs/institutes currently conducted in China.

### SSR genotyping, structure identification and genetic diversity analysis

Genomic DNA was extracted from sampled leaves using the CTAB method [22]. Seventy-seven previously published SSR markers [13–14, 21] were used for genotyping. PCR reaction, data collection and variation analysis were conducted following methods described in our previous studies [21]. We used parameters for STRUCTURE same as Jia et al. described (2013) [21]. To examine variations of elite varieties at the genomic level among decades and programs of release, total allele number was calculated with respect to these factors. Given the varied number of varieties per group, we used the random permutation procedure described in Fu et al. (2003) [23] to make comparisons between any two groups of elite varieties. FPTest (Fu permutation Test; Fu 2010) [24] was applied to calculate pairwise differences in allelic counts among groups of elite varieties and to test for statistical significance. All statistical data on cereal crops planting in China from the past sixty years were downloaded from http://data.stats.gov.cn/workspace/index?m=hgnd (National Bureau of Statistics of China). Foxtail millet cropping data from the last thirty years was downloaded from http://lib.cnki.net/cyfd/J149-N2005120141.html (Maintained by Ministry of Agriculture of the People’s Republic of China).

### Association analysis of agronomic traits

One hundred and ninety-three varieties released in the recent three decades were grown in Beijing (Geographic coordinate: N 39.93^0^, E 116.33^0^, Altitude 35m, in 2010 [E1] and 2011 [E2]), Anyang (Geographic coordinate: N 35.85^0^, E 114.07^0^, Altitude 75m, in 2011) [E3] and Changzhi (Geographic coordinate: N 36.65^0^, E 112.81^0^, Altitude 920m, in 2011) [E4] during summer growing seasons of foxtail millet. Eight agronomic characters including plant height (PH), panicle length (PL), peduncle length (PEL), panicle diameters (PD), stem diameters (SD), heading date (HD), panicle weight (PW) and grain weight of single panicle (GW) were measured for each accession. Five individual plants from each accession were randomly selected for the measurement. All association tests were conducted with the mixed linear model [25] using TASSEL [26] (“Trait Analysis by aSSociation, Evolution and Linkage”, a program written in Java for linkage disequilibrium statistics and association mapping). The population structure Q matrix was characterized using the software tool STRUCTURE [27] (A model-based clustering tool for inferring population structure through genotype data) using methods mentioned above. The relative kinship matrix K was constructed using SPAGeDi [28].

### Bottleneck determination and candidate gene selection

*Fst* (F statistics) values of microsatellite variations were used to detect genomic regions under selection pressures from landrace to elite varieties by using PowerMarker [29] (A computer program which performs statistical analysis of marker data), bootstraps times for all loci were 1,000 and the highest (>97.5%) SSRs were selected out for further analysis. SSR primer sequences were used for BLASTN analysis in Phytozome v9.1 (http://www.phytozome.net/search.php), and overlapping genes were determined as candidates.

## Results

### Genetic diversity of Chinese foxtail millet improved elite varieties

All seventy-seven SSR markers were successfully amplified from DNA of all elite variety accessions, and all markers were polymorphic across the 348 accessions. A total of 1376 alleles were detected. The average allele number per locus was 17.87, with a minimum of 5 and a maximum of 34. The average number of genotypes observed per locus was 24.20, with a minimum of 8 and a maximum of 80. For gene diversity, the average number of each locus was 0.82, ranging from 0.47 to 0.95. The average PIC (Polymorphic index content) value for markers was 0.80, ranging from 0.44 to 0.95. Heterozygosity per locus on average was 0.03, ranging from 0 to 0.40. The average homozygosity per line was greater than 95%, which indicated that, as expected given foxtail millet’s naturally selfing reproductive habit, most of the elite varieties used in this study were nearly inbred lines (**Table 2**).

**Table 2:** Genetic diversity of 348 improved foxtail millet elite varieties.

Sample	Allele No	Genotype No	Gene Diversity	Heterozygosity	PIC
Average	17.87	24.21	0.82	0.03	0.80
Range	5~34	8~80	0.47~0.95	0~0.40	0.45~0.95
Std.	8.14	11.80	0.10	0.04	0.12

### Population structure of Chinese elite varieties

**Fig 1:**
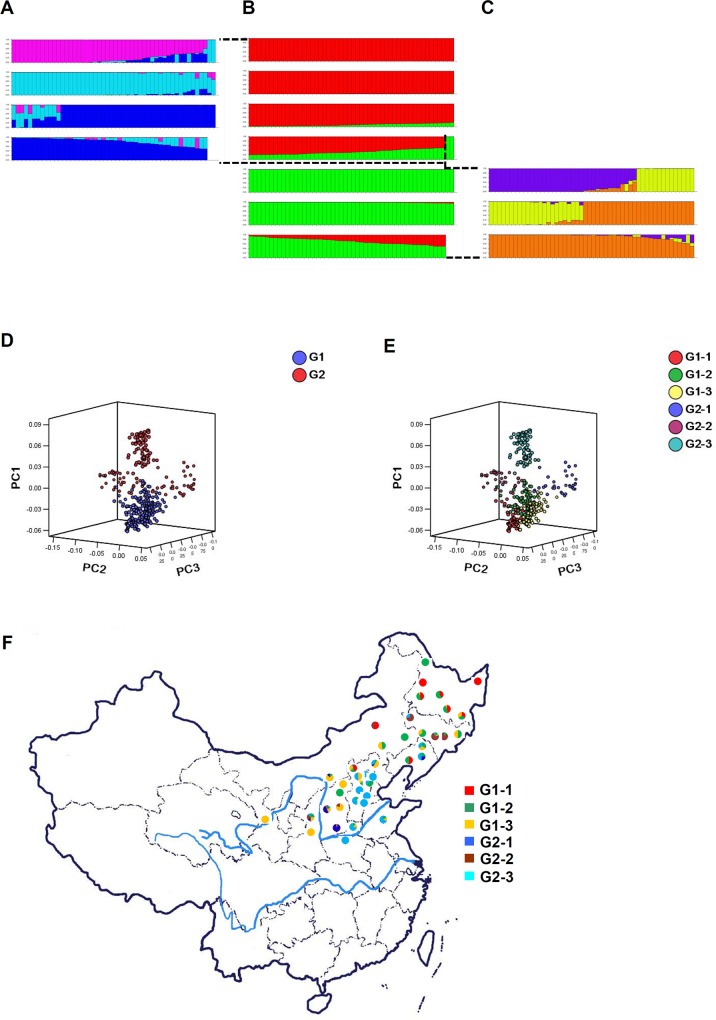
Inferred population structure of improved varieties. **(A)—(C):** Summary plot of estimates of Q. **(A)** Subclusters of group2 accessions; **(B)** Two groups inferred in this trial; **(C)** Subclusters of group1 accessions; **(D)** Differentiation of genotypes from clusters according to the first three principal components derived from 77 SSR markers diversity analysis; **(E)** Substructure within each main cluster inferred by STRUCTURE; **(F)** Distribution of the number of varieties allocated as respective designated subclusters within each province in North China.

Admixture model-based calculations were conducted by varying K from 1 to 10 with 20 iterations per K. When we ran the STRUCTURE simulations using all 348 accessions, the LnP(D) value increased with K from 1 to 10, but showed an evident knee at K = 2 (**S1A Fig**). This implied that there might be two divergent subpopulations. According to the second-order statistics developed for STRUCTURE [30] to estimate number of subpopulations, the optimal value of K = 2 whose delta K showed a peak was identified (**S1B Fig**). This result suggested that these foxtail millet varieties can be grouped into two populations, here designated as G1 and G2. For each inferred population, a second iteration of STRUCTURE analysis was conducted using the same approach. Our results indicated that G1 and G2 are both clearly divided into three (K = 3) subgroups (**Fig 1A–1C**). Here we designate the six subgroups identified as as G1-1, G1-2, G1-3, G2-1, G2-2, and G2-3.

The G1 cluster was comprised of varieties cultivated in spring-sowing eco-regions and a few lines in the summer-sowing regions but developed prior to the 1980s. Most elite varieties in the G1 group are not used in grain production currently. The G2 cluster was composed of elite varieties collected from the summer-sowing eco-regions, and were mostly developed after 1980s (**S1 Table**). A principal component analysis (PCA) was conducted to further assess the population subdivisions identified using STRUCTURE (**Fig 1D and 1E**). Plotting of the first three principal components (explaining 31.08%, 19.13% and 15.59% of the total marker variations, respectively) shows separation of the subpopulations inferred by STRUCTURE, and is also consistent with the neighbor-jointing tree of inferred subclusters (**S2 Fig**).

### Genetic variations among clusters and breeding periods

The genetic diversity was assessed per locus for each subgroup defined based on STRUCTURE, as well as for accessions grouped based on their released date (**Table 3**). Varieties in the G1 cluster possess higher levels of diversity, including higher number of alleles per locus, higher gene diversity and PIC values and greater numbers of cluster-specific alleles, compared to elite varieties grouped into G2. The diversity difference between subgroups and phylogenetic relationship among them can also be seen from a neighbor-jointing tree of STRUCTURE inferred subclusters within each topmost hierarchy cluster (**S3 Fig**). Pairwise estimates of *Fst* and genetic distance among six model-based subpopulations were also performed to infer genetic relationships among subgroups (**Table 4**). A neighbor-joining tree of the 348 varieties characterized in this study was concordant with the results of the STRUCTURE analysis (**S3 Fig**).

**Table 3:** Genetic diversities of improved elite varieties per locus grouped by inferred subclusters and periods.

Category	Sample size	Allele No.	Gene diversity	PIC	Specific allele	Percentage of total variation revealed by AMOVA	
						Among individuals within groups	Among alleles within individuals
**Inferred subclusters**							
**G1-1**	48	5.81	0.75	0.72	95	12.33	0.19
**G1-2**	64	11.29	0.77	0.74	74	16.53	0.49
**G1-3**	86	12.64	0.78	0.76	160	23.38	0.32
**G2-1**	36	6.07	0.60	0.56	17	7.46	0.26
**G2-2**	37	5.79	0.57	0.53	15	7.38	0.08
**G2-3**	77	7.71	0.61	0.57	33	16.48	0.29
**Improvement period**							
**1950s**	27	6.06	0.72	0.68	19	3.67	0.07
**1960s**	43	10.74	0.78	0.76	40	12.34	0.33
**1970s**	71	12.79	0.81	0.79	93	18.68	0.38
**1980s**	47	10.75	0.80	0.78	23	13.33	0.26
**1990s**	49	9.66	0.77	0.74	28	14.00	0.19
**2000s**	111	11.31	0.74	0.71	69	31.01	0.43

**Table 4:** Pairwise estimates of *Fst* and genetic distance among six model based subpopulations.

Subpop.	G1-1	G1-2	G1-3	G2-1	G2-2	G2-3
**G1-1**		0.0955	0.0929	0.2089	0.2121	0.2045
**G1-2**	0.3030		0.0711	0.1734	0.1974	0.1522
**G1-3**	0.2970	0.2215		0.1888	0.2370	0.1873
**G2-1**	0.4963	0.3538	0.3434		0.2585	0.2161
**G2-2**	0.4239	0.3722	0.4265	0.4420		0.2477
**G2-3**	0.4774	0.3318	0.4169	0.3567	0.3817	

*Fst* estimates appear above the diagonal and pairwise genetic distance appears below the diagonal.

Varieties from the same breeding programs and similar eco-environmental conditions tended to be more closely related (**Fig 1F**), as can be seen especially in the three subclusters in G2. Varieties released by breeding programs conducted in Shanxi and Jilin were dissimilar from most other lines, suggesting unique germplasm was incorporated into these breeding programs.

Varieties released in the 1970s had higher numbers of total alleles, gene diversity, PIC values and allele number per locus than varieties released in later decades (**Table 5**). A total of 272 alleles (20% of all alleles identified in the study) were identified as private alleles. Improved lines released in 1950s had the lowest allele count and the lowest genetic diversity in this analysis. In order to assess the significance of the differences observed between varieties released in different decades, FPTest was used to assess the statistical significance of differences in allele count between populations with different numbers of individuals. The results of this analysis indicated that allele number reductions of pairwise periods of 1990s/1970s, 1990s/1980s, and 2000s/1970s were significant at P<0.05, indicating a significantly loss of alleles accompanied progress in foxtail millet breeding progress in China.

**Table 5:** Allele count and variations of elite varieties released among last sixty years.

Period	Sample Size	Total allele number	1950s	1960s	1970s	1980s	1990s
**1950s**	27	467					
**1960s**	43	827	0.2309^$^				
**1970s**	71	985	0.0807	0.1601			
**1980s**	47	828	0.2361	0.2981	0.1793		
**1990s**	49	744	0.8750	0.0669	**0.0144** ^*^	**0.0293** ^*^	
**2000s**	111	871	0.9934	0.9919	**0.0016** ^**^	0.9993	0.9586

Pairwise difference were evaluated by *P* values of permutation test

$: *P* value of pairwised FPTest

**Fig 2:**
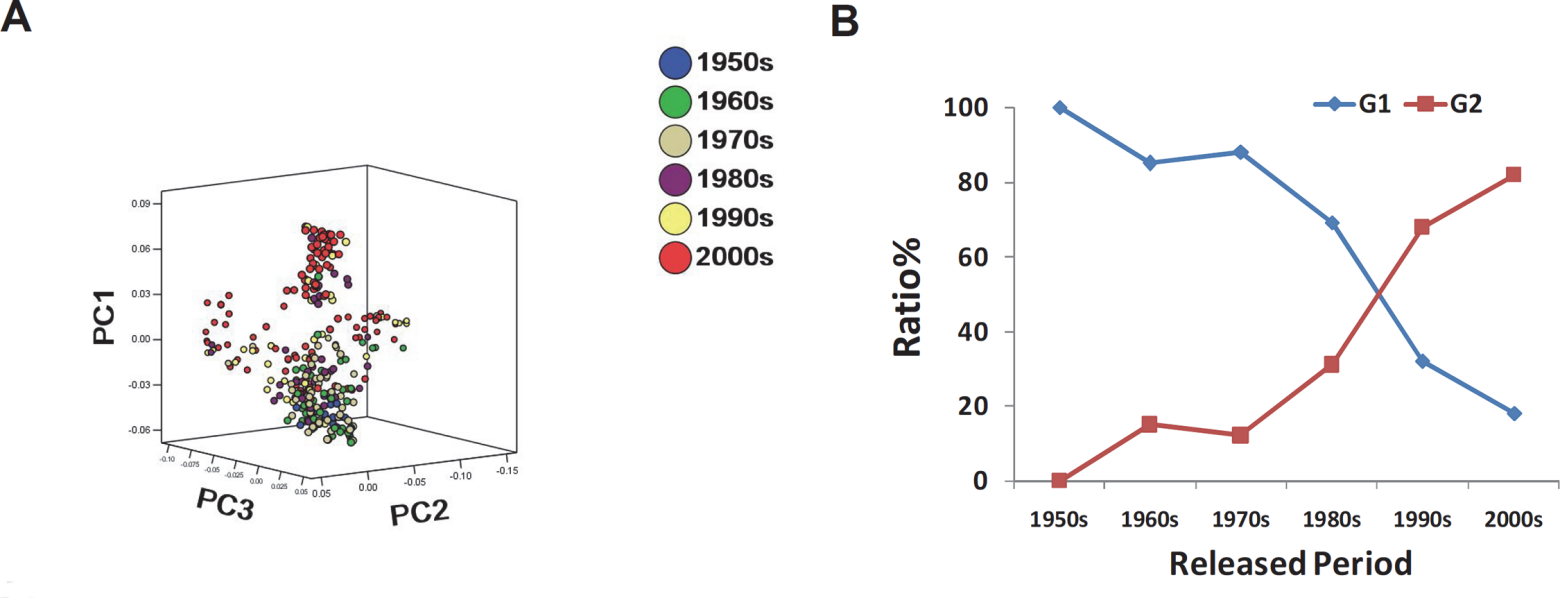
Transition of genetic structure of foxtail millet elite varieties in China. (**A**) Genetic diversification of varieties released in different periods as revealed by principal component analysis; (**B**) Relative variation of inferred sub-cluster proportions.

Associations of varieties were characterized using PCA (Principal Components Analysis) analysis (**Fig 2A**). By grouping varieties by their release date, an obvious transition in genetic constitution of foxtail millet varieties becomes apparent when varieties are grouped by release date (**Fig 2B**). In 1950s and 1960s, nearly all released varieties are spring-sowing ecotypes. Summer-sowing ecotypes begin to be released in 1970s and 1980s. In the recent two decades, the majority of newly released elite varieties are summer-sowing ecotypes, which was consistent with STRUCTURE inferred in Fig 1C. Data on planting of cereal crops over the past six decades in China (**S4A Fig**) shows a clear decline of production area devoted to foxtail millet accompanied an increase in the production area devoted to maize production in China. In the last thirty years, the geographical distribution of foxtail millet growing areas has shifted greatly (**S4B Fig**) which is correlated with the shift of germplasm foundation from primarily spring sowing ecotypes to summer sowing ecotypes in newly released improved varieties.

### Association mapping of agronomic characters

Association analysis using the 193 elite varieties in our dataset which were primarily released in the most three decades was used to identify the molecular basis of variation in key agronomic traits in foxtail millet. A total of 361 significant marker-phenotype correlations were observed (P<0.05) for eight morphological characters scored under four different experimental conditions (**S5 Fig**). Two-hundred-and-two (55.96%) of the significant marker/trait associations we detected were observed only in data from a single environmental condition, reinforcing the importance of genotype-by-environment interactions in the determining of morphological phenotypes in foxtail millet. Of the remaining marker/trait associations, 91 (25.21%) and 53 (14.68%) were identified simultaneously in two or three environments, respectively. Fifteen (4.15%) phenotype/marker associations were detected in all four environmental conditions, suggesting the alleles linked to these markers influence agronomic phenotypes in a largely environment independent manner (**Table 6**).

**Table 6:** Significant (P<0.05) QTLs conserved under four environmental conditions detected by association analysis.

Trait	SSR loci	Linkage group	E1		E2		E3		E4	
			R^2^	*P* value	R^2^	*P* value	R^2^	*P* value	R^2^	*P* value
PH	MPGA31	Chr.5	0.0364	0.0164	0.0649	0.0177	0.0906	0.0237	0.0328	0.0289
	x4	Chr.9	0.1068	2.02E-04	0.0271	0.0461	0.0445	0.0222	0.0379	5.15E-04
PL	b109	Chr.4	0.062	0.0407	0.0869	0.0494	0.1544	9.27E-04	0.1062	0.0296
	b182	Chr.1	4.49E-04	3.61E-05	0.1009	2.02E-04	0.1686	5.70E-04	0.1613	0.004
	b217	Chr.9	0.2313	1.36E-04	0.0677	0.002	0.2427	3.63E-04	0.276	3.91E-05
	b236	Chr.4	0.2169	0.0014	0.0923	0.0392	0.2116	0.0026	0.3149	9.34E-07
	b247	Chr.4	0.1264	4.75E-04	0.0936	0.0252	0.0962	0.0389	0.1274	0.0072
	b266	Chr.4	0.0537	0.0317	0.0835	0.001	0.0894	0.0337	0.106	0.016
	b269	Chr.9	0.1033	0.003	0.0661	0.0218	0.1781	3.42E-05	0.1642	0.0019
	p8	Chr.1	0.1413	1.41E-05	0.1159	0.0069	0.2409	2.63E-08	0.1778	5.34E-05
PEL	p2	Chr.4	0.1723	0.0036	0.1109	0.0113	0.1428	0.0216	0.0804	0.0402
PD	b123	Chr.7	0.0967	0.0282	0.0968	0.003	0.0713	4.45E-04	0.147	1.03E-05
	p98	Chr.3	0.0972	0.0081	0.0573	0.0443	0.0898	1.48E-06	0.1342	8.33E-06
HD	p44	Chr.9	0.0676	0.0396	0.0814	4.99E-04	0.0924	1.04E-04	0.0365	0.0154
SD	p29	Chr.7	0.2179	1.72E-05	0.055	0.0406	0.0619	0.0146	0.1163	3.02E-04

PH: Plant height; PL: Panicle length; PEL: Peduncle length; PD: Panicle diameters; HD: Heading date; SD: Stem diameters

## Discussion

### Diversity of Chinese foxtail millet elite varieties

In this study, the genetic diversity of a set of Chinese foxtail millet varieties, released over the past six decades, was analyzed using DNA microsatellite markers. The average number of alleles per locus was higher than previous reports by Jia et al. [13] and Liu et al. [15], which are 6.6 and 14.04 alleles per locus, respectively. The higher average allele number per locus observed in this study is likely the result of a larger sample size, covering a larger number of eco-regions and broader range of historical elite variety releases. Our previous study utilizing the same set of SSR markers identified an average of 20.9 alleles per locus in Chinese foxtail millet landraces and 33.5 alleles per locus in Chinese green foxtail accessions [14, 21]. This result is consistent with a loss of genetic diversity between landrace and elite gene pools as a result of a second genetic bottleneck during the development of modern foxtail millet elite varieties in China (**S6A and S6B Fig**). The loss of diversity during the domestication and improvement process of *S*. *italica* shown in this study agrees well with other ancient crop species, such as maize [31] and rice [32]. This implies that wild relatives of domesticated crop species could provide an abundance of essential and valuable alleles for future breeding programs, such as genes related to stress tolerance [33] and control of flowering time [34], which may have been lost during domestication.

### Structures of elite foxtail millet varieties in China

Analyses of phylogenetic relationships in this trial suggest that varieties released in China are clearly divided into two groups, which correspond to the summer and spring ecotypes. Heading date differentiations of the two groups under diverse environmental conditions also support this conjecture (**S7 Fig**). This result is concordant with the SNP result by Jia et al. (2013) [11], implying effective power of microsatellite markers in deciphering genetic structures of plant species with small size of genomes, as well as species with bigger genomes like maize and soybean [35–36]. Wang et al. (2012) [14] employed STRUCTURE to define four eco-regions among foxtail millet landrace accessions. In this study only two eco-types were defined among sampled foxtail millet elite varieties. This may due to the absence of a “South China” eco-type that identified in Wang et al. Based on our current study, no improved elite varieties were released in South China during last six decades, despite that prior to the 1950s, foxtail millet was a major crop in all the eco-regions of China and landraces were collected all over China [3, 14]. Secondly, landraces in the early-spring sowing eco-regions (ESR) and spring sowing regions (SR) have been replaced by elite varieties developed by pedigree selection (**S8A Fig**) from hybridization between local types and improved lines from summer sowing eco-regions. This probably led to the mixed genetic basis of elite varieties released in ESR and SR eco-regions of China that were generally designated as spring type in this study. Combined phylogenetic analysis of elite varieties and traditional landraces (**S6C and S6D Fig**) also illustrate that more landraces from Summer-sowing eco-regions (SSSR) have been utilized in variety breeding programs. For instance, the development of Yugu1 in the early 1980s in Henan province has greatly changed the breeding situation of foxtail millet. With strong resistance to lodging, higher grain yield and superior quality, Yugu1 was not only the dominant elite variety in agricultural production, but also served as the main parent for pedigree breeding in most breeding programs from then on, including those institutes located in spring sowing regions (**S8B Fig**). This meant many alleles from this summer sowing variety were incorporated into more recent spring sowing elite varieties and indeed, in this study we found that many elite varieties released after 1990s were in more genetically similar to the summer sowing type (**Fig 2B**).

### Inspiration of foxtail millet breeding strategy in the past and future

Although foxtail millet elite varieties still show high levels of diversity, the population structure and NJ (Neighbor Joining) classification presented here clearly indicate that the genetic diversity within individual breeding programs and institutes is decreasing (**Table 3**). Accessions released in the last two decades from the same breeding program are closely related and form distinct branches in the NJ tree (**S3 Fig**). Dissemination of beneficial alleles across different breeding programs has the potential to catalyze the development of greatly improved elite varieties, reversing the declining rate of yield gain observed for foxtail millet since the 1990s.

Foxtail millet landrace collections and comparisons were mostly conducted between the 1950s and the 1970s in China (**S8A Fig**). At that time, the dominant agricultural system was one year, one harvest, even in the North China Plain where the climate is such that two harvests per year are possible [37]. Most foxtail millet lines belonged to the spring sowing type and were adapted to sowing in late April or May. In the 1960s and 1970s, rapid population growth drove a transition to a one year two harvest system in the North China Plain [38], reflected by increasing multi-cropping index (MCI) in China. In a one year two harvest cropping system, foxtail millet is often sown in June after the harvest of a winter wheat crop. Varieties adapted to this cropping system were developed from the summer sowing ecotype in the late 1970s and to the 1980s in the North China Plain including Hebei, Shandong and Henan province. This transition from primarily spring sowing varieties to primarily summer sowing varieties revealed in this study was driven by the pursuing high MCI of cropping systems in China during the last six decades [39].

### QTLs controlling agronomic characters in foxtail millet

Association mapping analysis revealed that the majority of significant marker/phenotype correlations identified in this study were environment specific and acted only on a single trait, consistent with previous linkage analysis reports for foxtail millet [17, 19]. However, several associations were conserved across all environments and acted on multiple traits in foxtail millet, similar to the findings in other cereal crops [40–42]. All conserved associations detected in this trial (**Table 6**) are different from previous analysis [11] using world-wide collections, which might owe to different accessions, markers and statistical methods for STRUCTURE controlling that used in association studies. Trait associated markers that are conserved across multiple environmental conditions could be employed for Marker Assisted Selection (MAS) approaches to develop new foxtail millet elite varieties. These data also serve as a starting point for map-based cloning studies to identify specific genes responsible for the observed variation. This is particularly crucial for peduncle length, a trait for which the molecular basis remains unclear in grass crops. For panicle length, two markers (b217, Chr.9; b236, Chr.4) significantly associated with the trait explained over 20% phenotypic variance (**Table 6**) under three of the four environmental conditions and may represent valuable genomic regions contributed to this important morphological and grain-yield related agronomic trait.

An *F*-test between foxtail millet elite varieties and landraces revealed 11 SSR loci that had significantly (>97.5%) diversified between these two gene pools, owing to the long period of breeding selection or local adaptation (**S9 Fig**). Two loci were localized in gene-coding regions (*Si017865m* and *Si016673m*), which are potentially important genes involved in different metabolic pathway or have played vital roles in foxtail millet improvement. All 11 genomic regions under selection were co-localized with significantly association loci controlling agronomically important traits in foxtail millet (**S2 Table**). These may be vital loci for morphological improvement of agronomic traits in foxtail millet. Results of the co-localization of selective sweep regions detected by SSRs and GWAS in this study implies that breeding of elite varieties of foxtail millet in China has been mainly focused on selection of multiple diverse minor-effect loci controlling different agronomic traits during long term breeding for improved varieties. This is dissimilar from domestication related selection where there can be rapid selection for large effect alleles which change plant morphology [34]. It can be inferred that much of the process of variety improvement in foxtail millet might be due to the roles of gene-by-gene interaction and gene-by-environment effects in shaping the rate of phenotypic changes of crop improvement rather than single gene changes, although this remains to be verified in the future.

Raw data created in this trial could be found in supplemental materials as S3 Table.

## Supporting Information

S1 Fig Determination of optimal value of K for STRUCTURE analysis. **(A)** Proper K inferred through the ad hoc procedure described in Pritchard et al. (2000); **(B)** The second order of statistics of Delta K based on methods developed by Evanno et al. (2005) for optimal K value determination.
(TIF)

S2 Fig Neighbor-jointing tree of 348 varieties characterized using SSRs. (TIF)

S3 Fig Determination of proper value of K for substrucutring and Neighbor-jointing tree of STRUCTURE inferred subclusters within each topmost hierarchy cluster. **(A)** and **(B)**: Proper K identification by LnP(D) and delta K of G1; **(C)** and **(D)**: Proper K identification by LnP(D) and delta K of G2; Phylogenic relationships among varieties **(E)** and subclusters **(F)** were indicated by colored lines.
(TIF)

S4 Fig Planting configurations of cereal crops in China. (**A**) Cropping area transitions of main cereal crops including rice, wheat, maize, foxtail millet, sorghum and barley during last six decades; (**B**) Changing of planting distributions of foxtail millet in China during last thirty years.
(TIF)

S5 Fig Association mapping of QTLs controlling agronomically important traits in foxtail millet. **(A)** Venn plot of the numbers of significant (P<0.05) QTLs detected in this trial among four environments; **(B)** Relationship between number of significantly associated SSR markers and agronomic traits under diverse environmental conditions; **(C)** Relationship between numbers of traits associated with the same marker and amount of SSR loci.
(TIF)

S6 Fig Selective bottlenecks during domestication and improvement processes of *Setaria italica* in China. Allele and genotype no. **(A)** and gene diversity, PIC values and heterozygosity **(B)** per locus detected in three panels of gene pools of *Setaria* identified using SSRs. Microsatellite diversity data were collected from Wang et al. (2012), Jia et al. (2013) and this study, which were analyzed using the same set of SSRs in the same lab; **(C)** Phylogenetic relationships between landraces (Blue) (Wang et al., 2012) and elite varieties (Green) sampled in this trial; **(D)** Phylogenetic analysis of four subclusters defined in landraces (Wang et al., 2012) and subgroups inferred in this study.
(TIF)

S7 Fig Heading date of group1 and group2 accessions inferred in this trial. (TIF)

S8 Fig Variation of breeding approaches (A) and cultivating area (B) of elite foxtail millet varieties released during last six decades in China. (TIF)

S9 Fig Distribution of *Fst* values of SSR loci between two gene pools of landraces and elite varieties. Bootstraps across loci were repeated for 1000 times and the 97.5% up border is indicated by broken lines.
(TIF)

S1 Table List of varieties sampled in this trial according to subclusters inferred from STRUCTURE. (DOC)

S2 Table Associations of genomic regions under selection (>97.5%) during modern improvement process and agronomic traits detected by GWAS analysis (P<0.05) in foxtail millet. (DOC)

S3 Table Raw sequencing data of PCR products created for analysis in this study. (XLS)

## References

[1] Lu H, Zhang J, Liu KB, Wu N, Li Y, Zhou K, et al Earliest domestication of common millet (*Panicum miliaceum*) in east Asia extended to 10,000 years ago. Proceedings of the National Academy of Sciences, USA 2009; 106: 7367–7372. 10.1073/pnas.0900158106 PMC2678631 19383791

[2] Yang X, Wan Z, Perry L, Lu H, Wang Q, Zhao C, et al Early millet use in northern china. Proceedings of the National Academy of Sciences, USA 2012; 109: 3726–3730. 10.1073/pnas.1115430109 22355109 PMC3309722

[3] Diao X. Current status of foxtail millet production in China and future development direction In: Diao X. editors. Foxtail millet production and research system in China. Beijing: China Agricutural Science and Technology Press; 2011 pp. 20–30.

[4] Diao X, Schnable J, Bennetzen JL, Li J. Initiation of Setaria as a model plant. Frontiers of Agricultural Science and Engineering 2014; 1(1): 16–20.

[5] Li P, Brutnell TP. *Setaria viridis* and *Setaria italica*, model genetic systems for the Panicoid grasses. Journal of Experimental Botany 2011; 62: 3031–3037. 10.1093/jxb/err096 21459768

[6] Karki S, Rizal G, Quick WP. Improvement of photosynthesis in rice (*Oryza sativa L*.) by inserting the C4 pathway. Rice 2013; 6: 28 10.1186/1939-8433-6-28 24280149 PMC4883725

[7] Doust AN, Kellogg EA, Devos KM, Bennetzen JL. Foxtail millet: A sequence-driven grass model system. Plant Physiology 2009; 149: 137–141. 10.1104/pp.108.129627 19126705 PMC2613750

[8] Lata C, Gupta S, Prasad M. Foxtail millet: a model crop for genetic and genomic studies in bioenergy grasses. Critical Reviews in Biotechnology 2012; 33: 328–343. 10.3109/07388551.2012.716809 22985089

[9] Bennetzen JL, Schmutz J, Wang H, Percified R, Hawkins J, Pontaroli A C, et al Reference genome sequence of the model plant Setaria. Nature Biotechnology 2012; 30: 556–561. 10.1038/nbt.2196 22580951

[10] Zhang G, Liu X, Quan Z, Cheng S, Xu X, Pan S, et al Genome sequence of foxtail millet (*Setaria italica*) provides insights into grass evolution and biofuel potential. Nature Biotechnology 2012; 30: 549–554. 10.1038/nbt.2195 22580950

[11] Jia G, Huang X, Zhi H, Zhao Y, Zhao Q, Li W, et al A haplotype map of genomic variations and genome-wide association studies of agronomic traits in foxtail millet (*Setaria italica*). Nature Genetics 2013; 45: 957–961. 10.1038/ng.2673 23793027

[12] Wang T, Du R, Chen H, Darmency H, Fleury A. A new way of using herbicide resistant gene on hybrid utilization in foxtail millet. SCIENTA AGRICULTURA SINCA 1996; 29: 96.

[13] Jia X, Zhang Z, Liu Y, Zhang C, Shi Y, Song Y, et al Development and genetic mapping of SSR markers in foxtail millet [*Setaria italica* (L.) P.Beauv.]. Theoretical and Applied Genetics 2009; 118: 821–829. 10.1007/s00122-008-0942-9 19139840

[14] Wang C, Jia G, Zhi H, Niu Z, Chai Y, Li W, et al Genetic diversity and population structure of Chinese foxtail millet [*Setaria italica* (L.) Beauv.] landraces. G3 (Genes, Genomes, Genetics) 2012; 2: 769–777. 10.1534/g3.112.002907 22870400 PMC3385983

[15] Liu Z, Bai G, Zhang D, Zhu C, Xia X, Cheng R, et al Genetic diversity and population structure of elite foxtail millet [*Setaria italica* (L.) P. Beauv.] germplasm in China. Crop Science 2011; 51: 1655–1663.

[16] Vetriventhan M, Upadhyaya HD, Anandakumar CR, Senthilvel S, Parzies HK, Bharathi A, et al Assessing genetic diversity, allelic richness and genetic relationship among races in ICRISAT foxtail millet core collection. Plant Genetic Resources 2012; 1(10): 214–223.

[17] Doust AN, Devos KM, Gadberry M, Gale MD, Kellogg EA. Genetic control of branching in the foxtail millet. Proceedings of the National Academy of Sciences, USA 2004; 101: 9045–9050. 15184666 10.1073/pnas.0402892101 PMC428470

[18] Mauro-Herrera M, Wang X, Barbier H, Brutnell TP, Devos KM, Doust AN. Genetic control and comparative genomic analysis of flowering time in Setaria (Poaceae). G3 (Genes, Genomes, Genet) 2013; 3: 283–295. 10.1534/g3.112.005207 23390604 PMC3564988

[19] Doust AN, Devos KM, Gadberry M, Gale MD, Kellogg EA. The genetic basis for inflorescence variation between foxtail and green millet (Poaceae). Genetics 2005; 169: 1659–1672. 15654107 10.1534/genetics.104.035543 PMC1449545

[20] Gupta S, Kumari K, Muthamilarasan M, Parida SK, Prasad M. Population structure and association mapping of yield contributing agronomic traits in foxtail millet. Plant Cell Reports 2014; 33: 881–893. 10.1007/s00299-014-1564-0 24413764

[21] Jia G, Shi S, Wang C, Niu Z, Chai Y, Zhi H, et al Molecular diversity and population structure of Chinese green foxtail [*Setaira viridis* (L.) Beauv.] revealed by microsatellite analysis. Journal of Experimental Botany 2013; 64: 3645–3655. 10.1093/jxb/ert198 23956411 PMC3745726

[22] Doyle JJ. DNA protocol for plants CTAB total DNA isolation In: Hewitt GM, editors. Molecular Techniques in Taxonomy. Berlin: Springer; 1991 pp. 283–293.

[23] Fu YB, Peterson GW, Scoles G, Rossnagel B, Schoen DJ, Richards KW. Allelic diversity changes in 96 Canadian oat cultivars released from 1886 to 2001. Crop Science 2003; 43: 1989–1995.

[24] Fu Y. FPTEST: a SAS routine for testing differences in allelic count. Molecular Ecology Resources 2010; 10: 389–392. 10.1111/j.1755-0998.2009.02752.x 21565035

[25] Yu J, Pressoir G, Briggs WH, Bi IV, Yamasaki M, Doebley JF, et al A unified mixed-model method for association mapping that accounts for multiple levels of relatedness. Nature Genetics 2006; 38: 203–208. 16380716 10.1038/ng1702

[26] Bradbury PJ, Zhang Z, Kroon DE, Casstevens TM, Ramdoss Y, Buckler ES. TASSEL: Software for association mapping of complex traits in diverse samples. Bioinformatics 2007; 23: 2633–2635. 17586829 10.1093/bioinformatics/btm308

[27] Pritchard JK, Stevens M, Donnelly P. Inference of population structure using multilocus genotype data. Genetics 2000; 155: 945–959. 10835412 10.1093/genetics/155.2.945 PMC1461096

[28] Hardy OJ, Vekemans X. SPAGeDi: a versatile computer program to analyse spatial genetic structure at the individual or population levels. Molecular Ecology Notes 2002; 2(4): 618–620.

[29] Liu K, Muse SV. PowerMarker: integrated analysis environment for genetic marker data. Bioinformatics 2005; 21: 2128–2129. 15705655 10.1093/bioinformatics/bti282

[30] Evanno G, Regnaut S, Goudet J. Detecting the number of clusters of individuals using the software STRUCTURE: a simulation study. Molecular Ecology 2005; 14: 2611–2620. 15969739 10.1111/j.1365-294X.2005.02553.x

[31] Hufford MB, Xu Xun, Heerwaarden JV, Pyhäjärvi T, Chia J, Cartwright RA, et al Comparative population genomics of maize domestication and improvement. Nature Genetics 2012; 44: 808–811. 10.1038/ng.2309 22660546 PMC5531767

[32] Huang X, Kurata N, Wei X, Wang ZX, Wang A, Zhao Q, et al A map of rice genome variation reveals the origin of cultivated rice. Nature 2012; 490: 497–501. 10.1038/nature11532 23034647 PMC7518720

[33] Qie L, Jia G, Zhang W, Schnable J, Shang Z, Li W, et al Mapping of quantitative trait locus (QTLs) that contribute to germination and early seedling drought tolerance in the interspecific cross *Setaria italica*×*Setaria viridis* . PLOS ONE 2014; 9(7): e101868 10.1371/journal.pone.0101986 25033201 PMC4102488

[34] Doust AN, Lukens L, Olsen KM, Mauro-Herrera M, Meyer A. Beyond the single gene: How epistasis and gene-by-environment effects influence crop domestication. Proceedings of the National Academy of Sciences, USA 2014; 10.1073/pnas.1308940110 PMC4035984 24753598

[35] Matsuoka Y, Vigouroux Y, Goodman MM, G. JS, Buckler E, Doebley J. A single domestication for maize shown by multilocus microsatellite genotyping. Proceedings of the National Academy of Sciences, USA 2002; 99: 6080–6084. 11983901 10.1073/pnas.052125199 PMC122905

[36] Li Y, Li W, Zhang C, Yang L, Chang R, Gaut BS, Qiu L. Genetic diversity in domesticated soybean (*Glycine max*) and its wild progenitor (*Glycine soja*) for simple sequence repeat and single-nucleotide polymorphism loci. New Phytologist 2010; 188: 242–253. 10.1111/j.1469-8137.2010.03344.x 20618914

[37] Piao S, Ciais P, Huang Y, Shen Z, Peng S, Li J, et al The impacts of climate change on water resources and agriculture in China. Nature 2010; 467: 43–51. 10.1038/nature09364 20811450

[38] Zhu H, Li X, Xin L. Intensity change in cultivated land use in China and its policy implications. Journal of Natural Resources 2007; 22: 907–915.

[39] Xin L, Li X, Zhu H, Tan M. China’s potential of grain production due to changes in agricultural land utilization in recent years. Chinese Geographical Science 2009; 19(2): 97–103.

[40] Jiao Y, Wang Y, Xue D, Wang J, Yan M, Liu G, et al Regulation of OsSPL14 by OsmiR156 defines ideal plant architecture in rice. Nature Genetics 2010; 42: 541–544. 10.1038/ng.591 20495565

[41] Miura K, Ikeda M, Matsubara A, Song X, Ito M, Asano K, et al OsSPL14 promotes panicle branching and higher grain productivity in rice. Nature Genetics 2010; 42: 545–549. 10.1038/ng.592 20495564

[42] Xue W, Xing Y, Weng X, Zhao Y, Tang W, Wang L, et al Natural variation in Ghd7 is an important regulator of heading date and yield potential in rice. Nature Genetics 2008; 40: 761–767. 10.1038/ng.143 18454147

