# Supplementary figures and images for: Microsatellite Variations of Elite Setaria Varieties Released during Last Six Decades in China
Source: PLoS One. 2015 May 1;10(5):e0125688. doi: 10.1371/journal.pone.0125688 (PMC4416935; doi:10.1371/journal.pone.0125688)

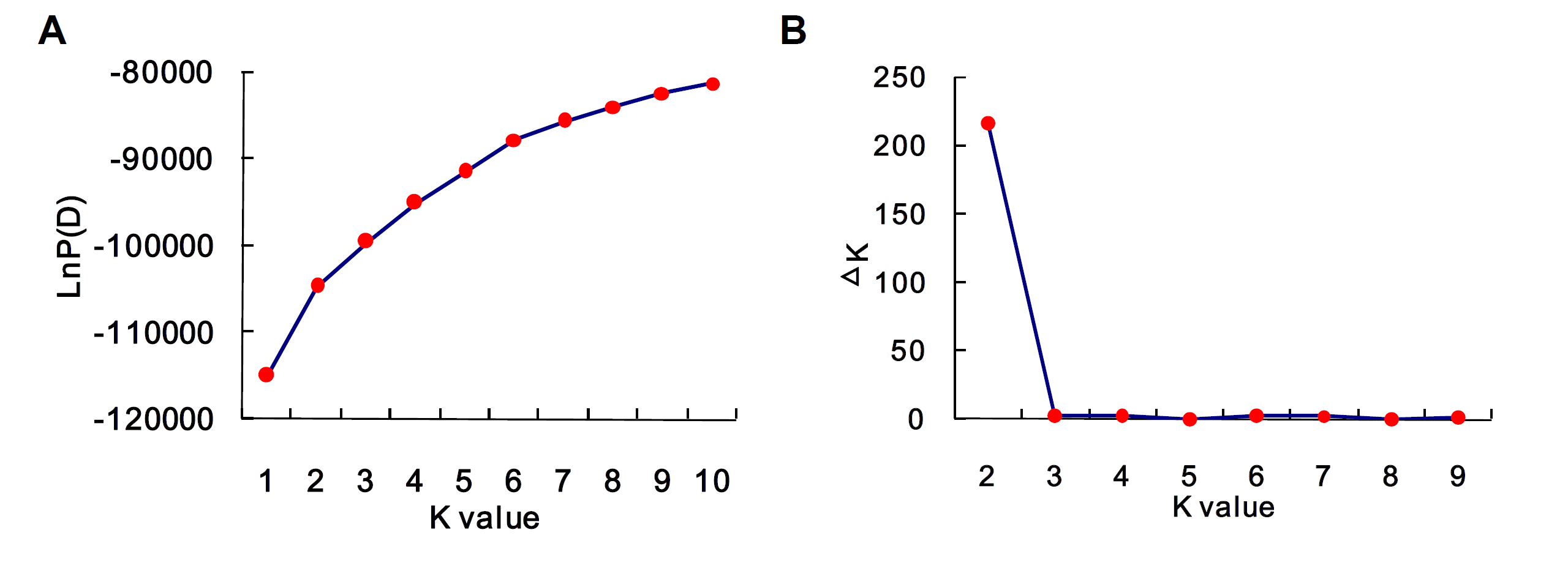

Supplement: S1 Fig — (A) Proper K inferred through the ad hoc procedure described in Pritchard et al. (2000); (B) The second order of statistics of Delta K based on methods developed by Evanno et al. (2005) for optimal K value determination. (TIF) [file pone.0125688.s001.tif]

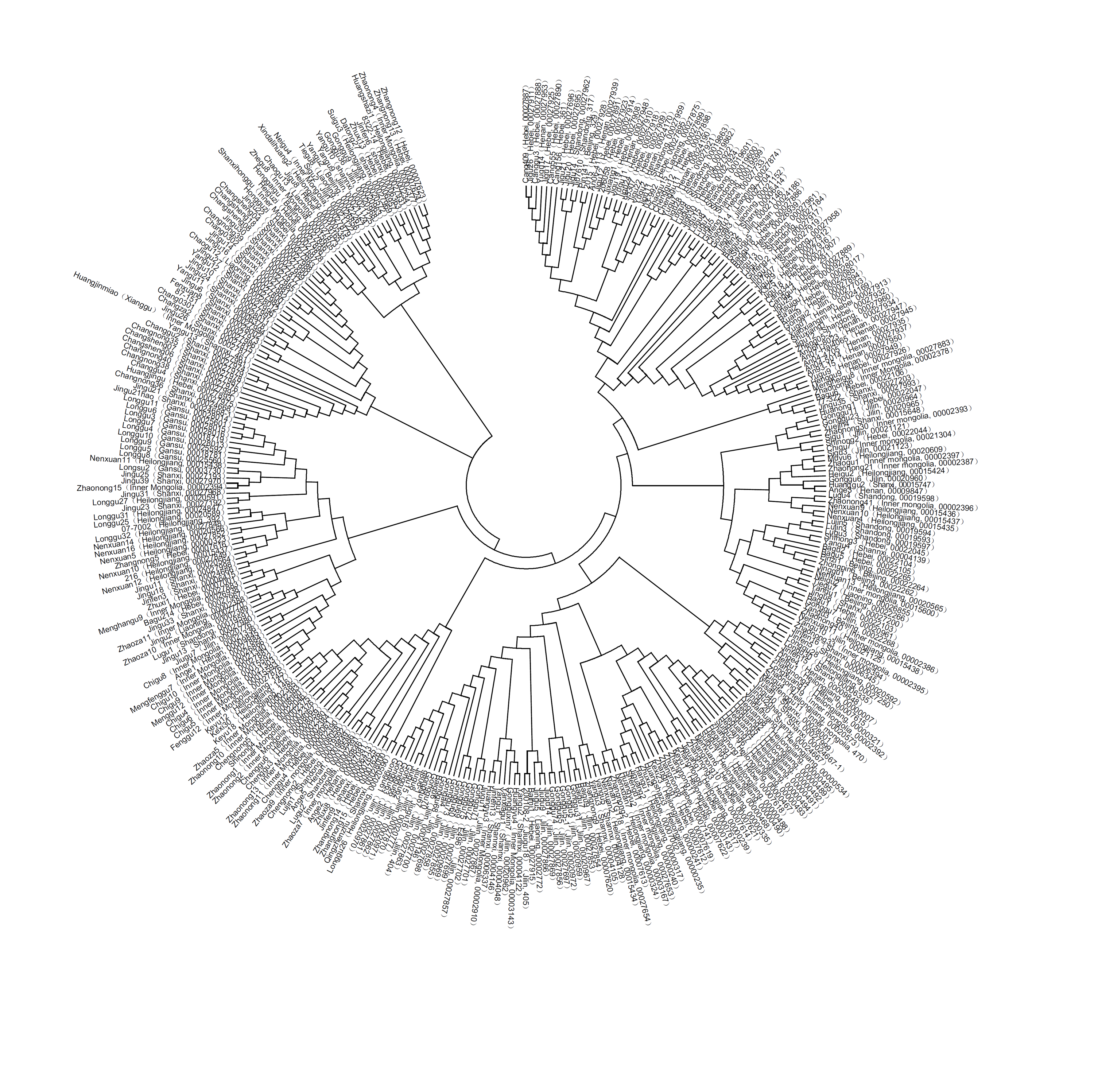

Supplement: S2 Fig — (TIF) [file pone.0125688.s002.tif]

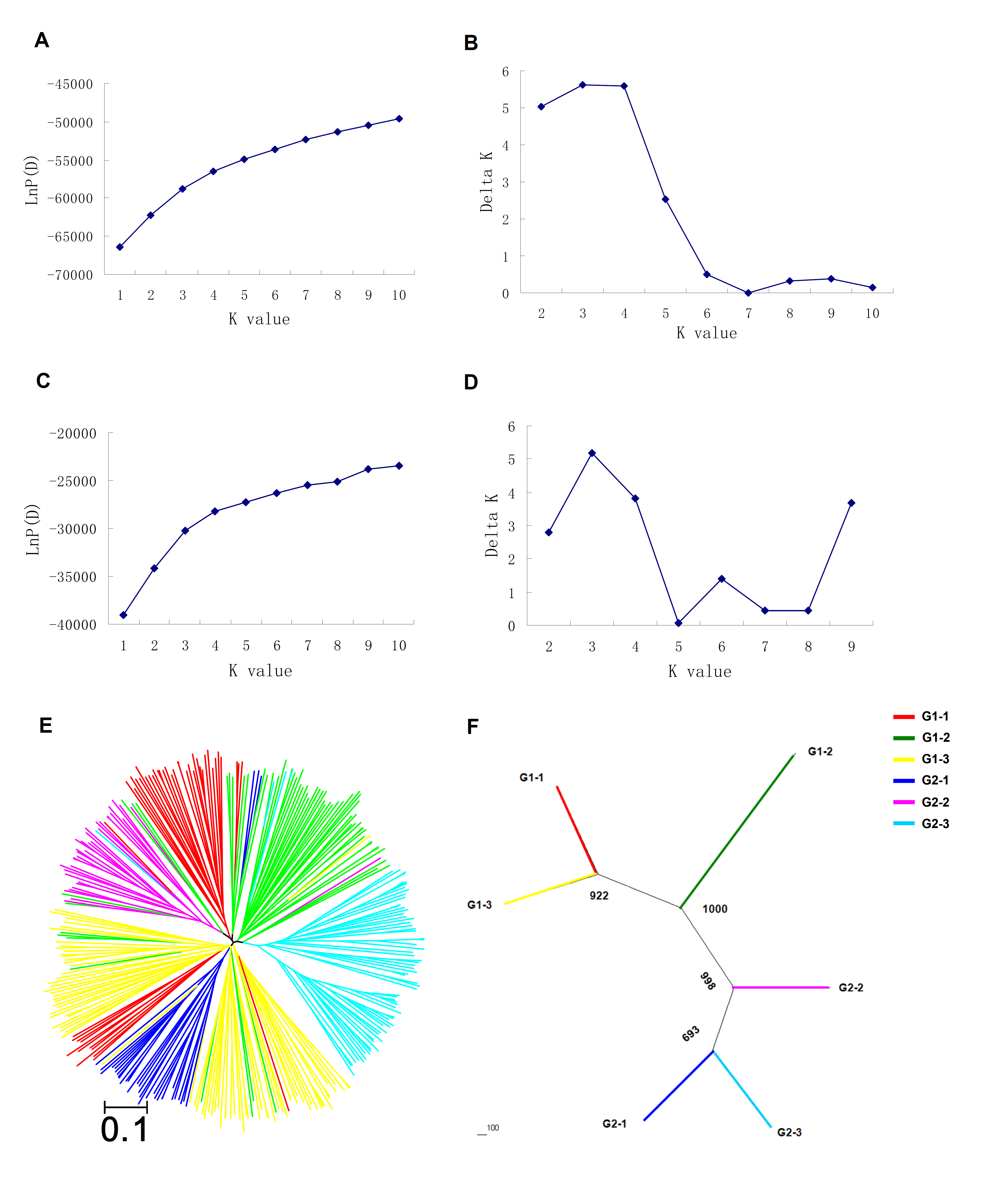

Supplement: S3 Fig — (A) and (B): Proper K identification by LnP(D) and delta K of G1; (C) and (D): Proper K identification by LnP(D) and delta K of G2; Phylogenic relationships among varieties (E) and subclusters (F) were indicated by colored lines. (TIF) [file pone.0125688.s003.tif]

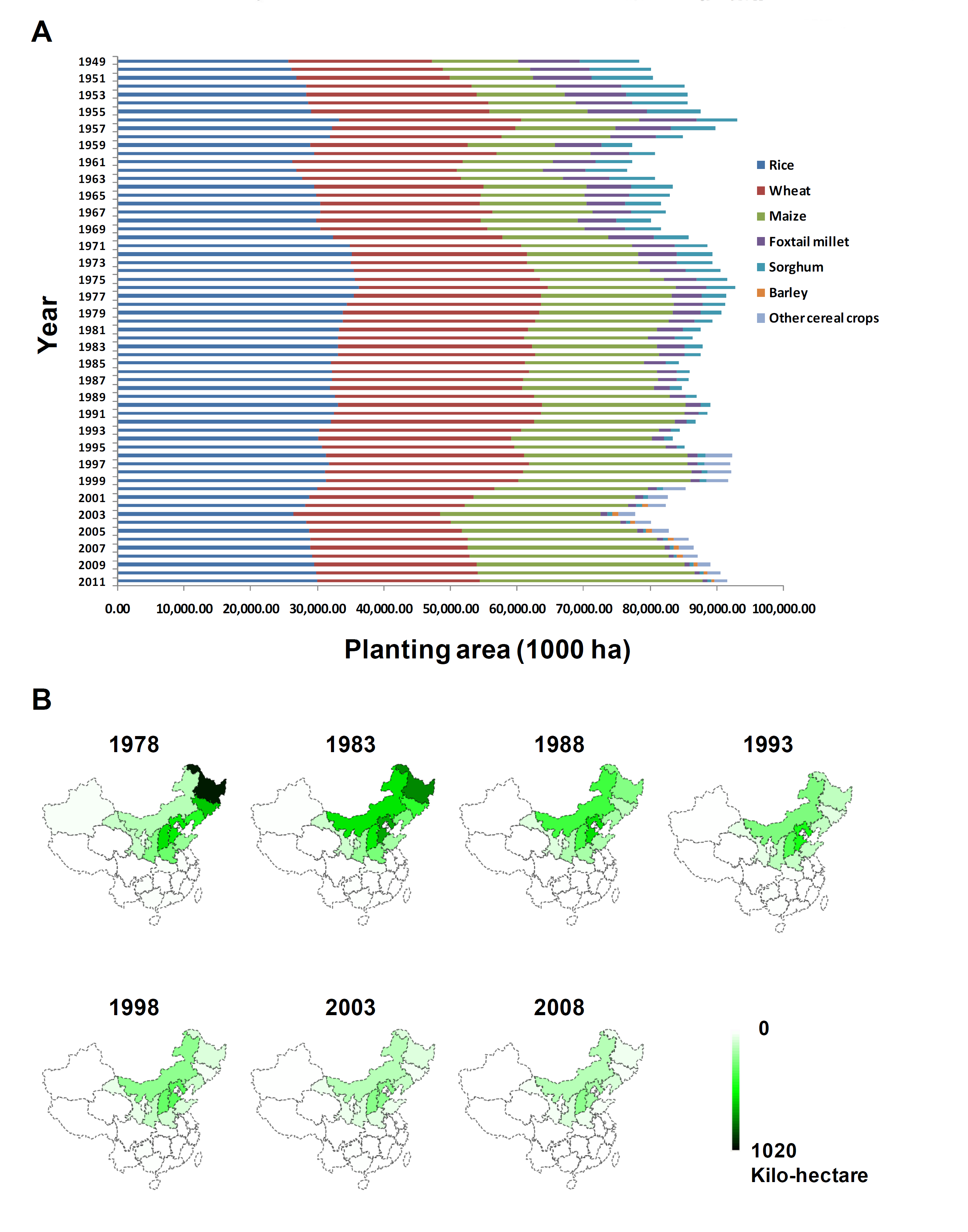

Supplement: S4 Fig — (A) Cropping area transitions of main cereal crops including rice, wheat, maize, foxtail millet, sorghum and barley during last six decades; (B) Changing of planting distributions of foxtail millet in China during last thirty years. (TIF) [file pone.0125688.s004.tif]

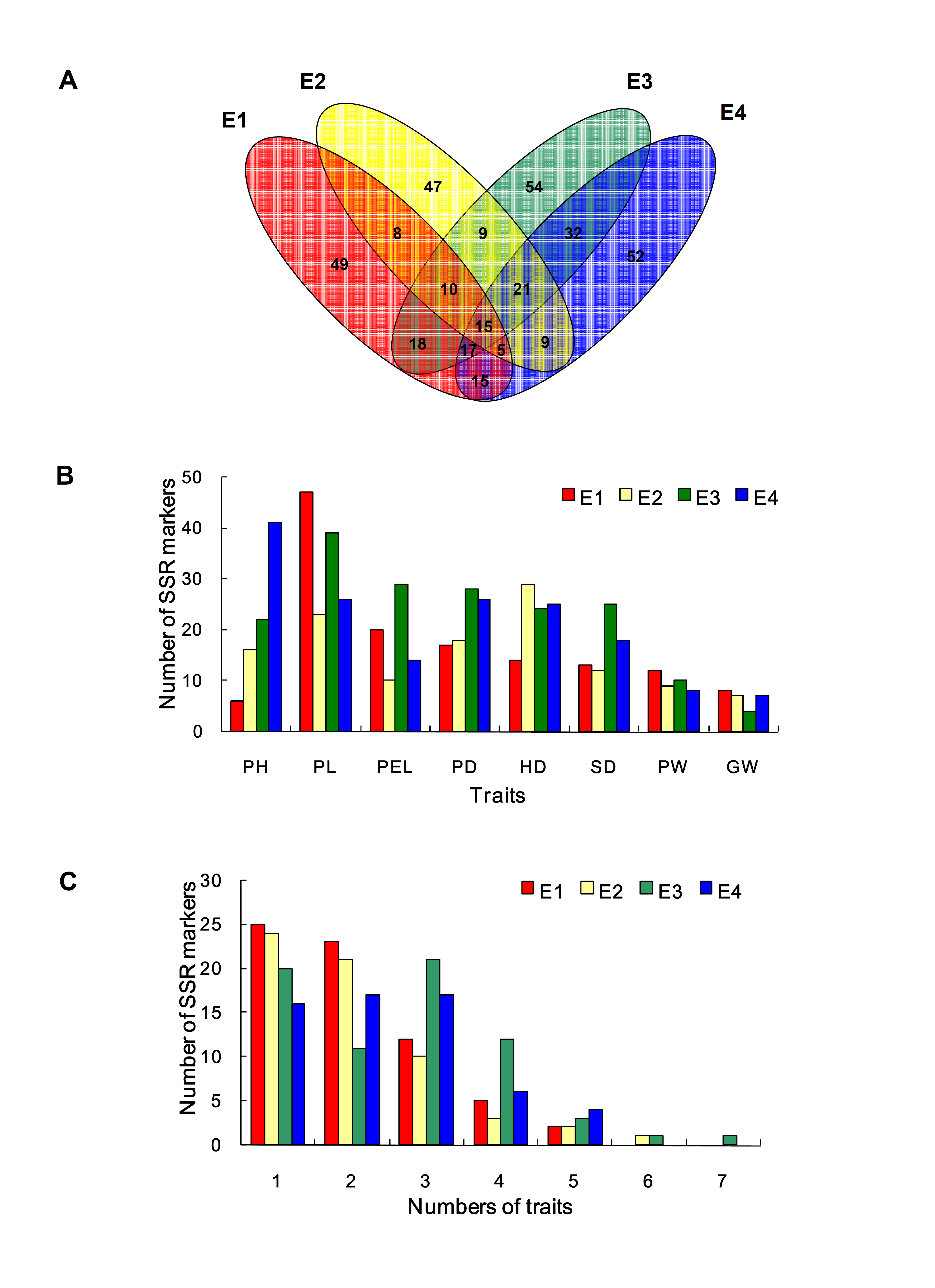

Supplement: S5 Fig — (A) Venn plot of the numbers of significant (P<0.05) QTLs detected in this trial among four environments; (B) Relationship between number of significantly associated SSR markers and agronomic traits under diverse environmental conditions; (C) Relationship between numbers of traits associated with the same marker and amount of SSR loci. (TIF) [file pone.0125688.s005.tif]

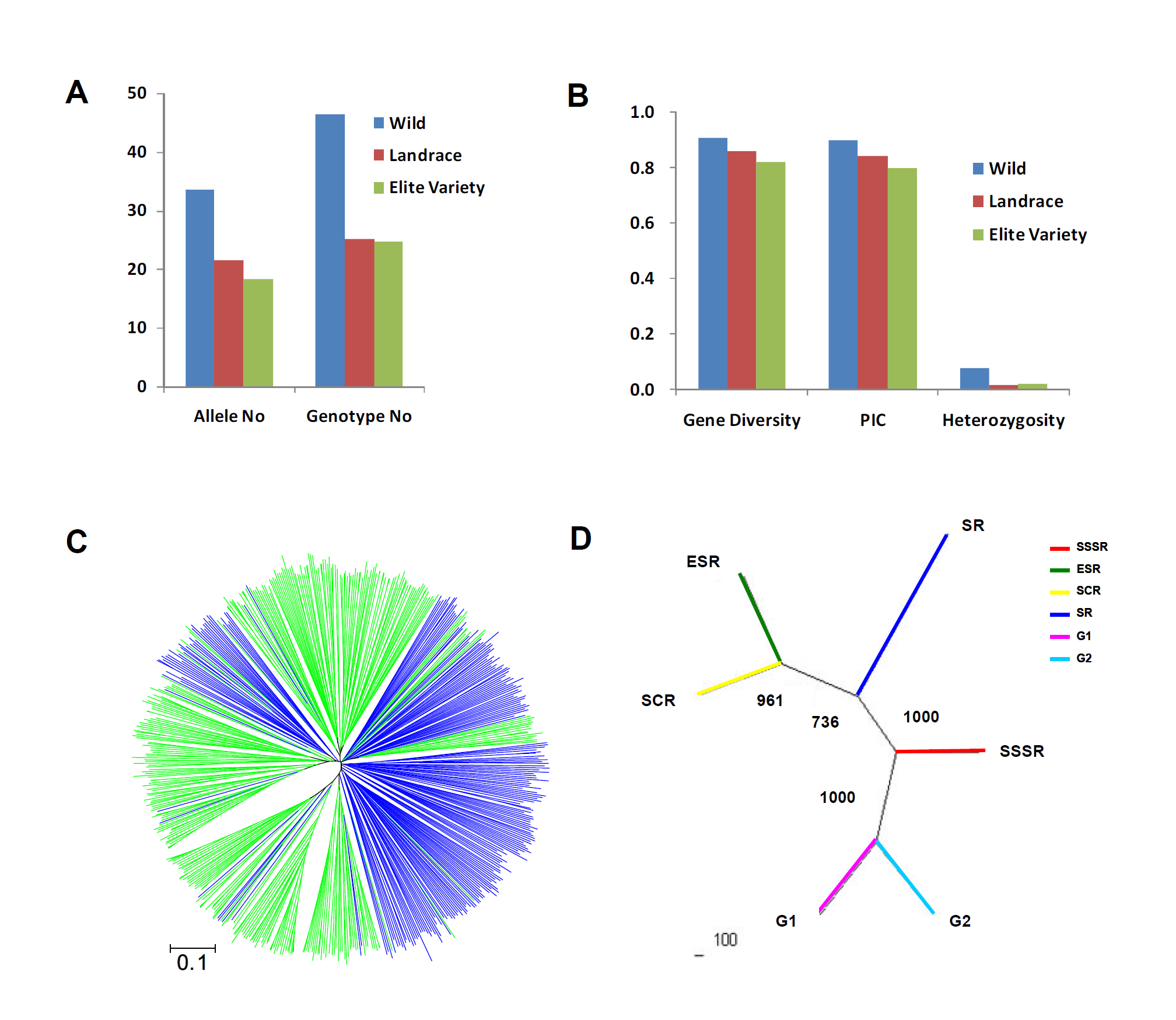

Supplement: S6 Fig — Allele and genotype no. (A) and gene diversity, PIC values and heterozygosity (B) per locus detected in three panels of gene pools of Setaria identified using SSRs. Microsatellite diversity data were collected from Wang et al. (2012), Jia et al. (2013) and this study, which were analyzed using the same set of SSRs in the same lab; (C) Phylogenetic relationships between landraces (Blue) (Wang et al., 2012) and elite varieties (Green) sampled in this trial; (D) Phylogenetic analysis of four subclusters defined in landraces (Wang et al., 2012) and subgroups inferred in this study. (TIF) [file pone.0125688.s006.tif]

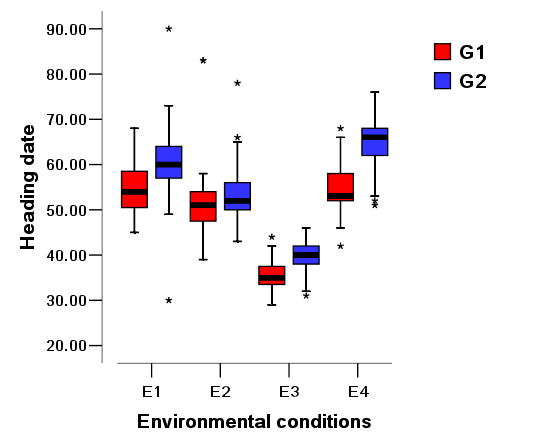

Supplement: S7 Fig — (TIF) [file pone.0125688.s007.tif]

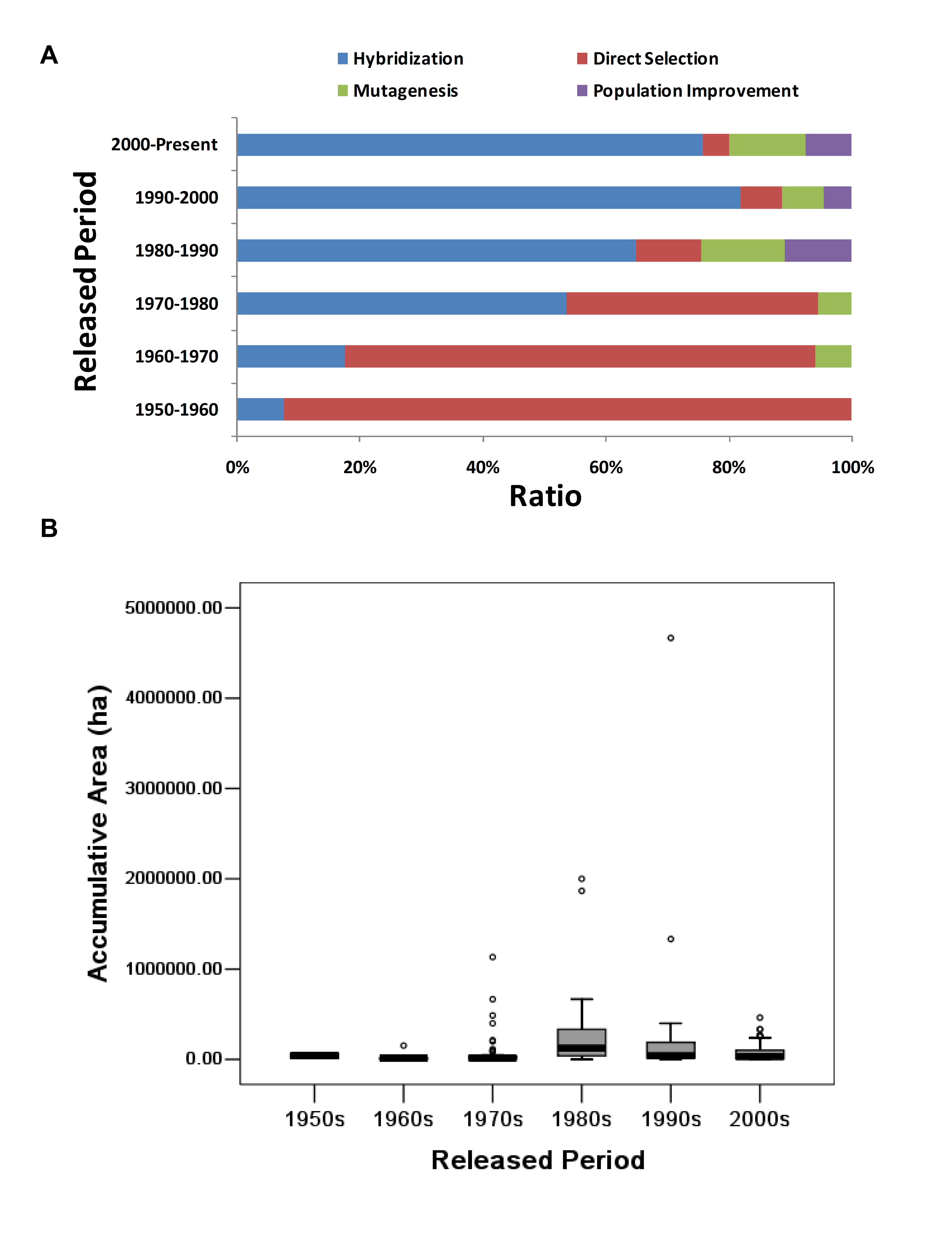

Supplement: S8 Fig — (TIF) [file pone.0125688.s008.tif]

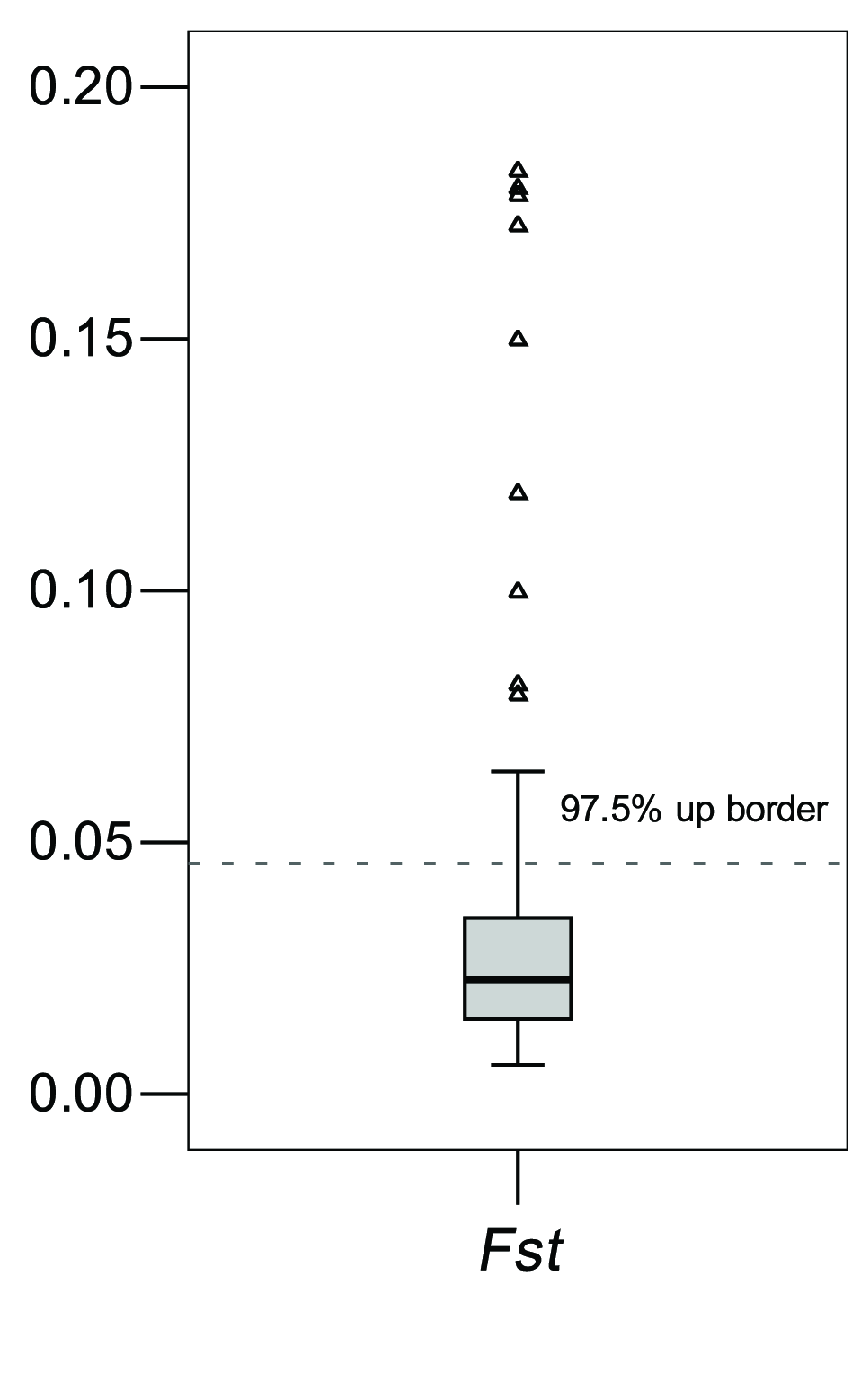

Supplement: S9 Fig — Bootstraps across loci were repeated for 1000 times and the 97.5% up border is indicated by broken lines. (TIF) [file pone.0125688.s009.tif]
