# Supplementary material for: Microsatellite Variations of Elite Setaria Varieties Released during Last Six Decades in China
Source: PLoS One. 2015 May 1;10(5):e0125688. doi: 10.1371/journal.pone.0125688 (PMC4416935; doi:10.1371/journal.pone.0125688)
Supplement: S1 Table — (DOC) [file pone.0125688.s010.doc]

**Table S1** List of varieties sampled in this trial according to subclusters inferred from STRUCTURE

| Sample ID | Variety name | Province | Breeding program | Released period | Gene bank No. |
| --- | --- | --- | --- | --- | --- |
| **G1-1** |  |  |  |  |  |
| 1 | Longgu32 | Heilongjiang | Institute of breeding, Heilongjiang academy of agriculture | 2000s | 00027848 |
| 2 | Longgu25 | Heilongjiang | Institute of breeding, Heilongjiang academy of agriculture | 1980s | 00020589 |
| 3 | Longgu30 | Heilongjiang | Institute of breeding, Heilongjiang academy of agriculture | 1990s | 00027250 |
| 4 | Longgu31 | Heilongjiang | Institute of breeding, Heilongjiang academy of agriculture | 2000s | 00027847 |
| 5 | 216 | Heilongjiang | Institute of breeding, Heilongjiang academy of agriculture | 2000s | 00027849 |
| 6 | 07-7002 | Heilongjiang | Institute of breeding, Heilongjiang academy of agriculture | 2000s | 392 |
| 7 | Longgu28 | Heilongjiang | Institute of breeding, Heilongjiang academy of agriculture | 1980s | 00020592 |
| 8 | Nenxuan10 | Heilongjiang | Institute of nenjiang, Heilongjiang academy of agriculture | 1970s | 00015437 |
| 9 | Nenxuan16 | Heilongjiang | Institute of nenjiang, Heilongjiang academy of agriculture | 2000s | 00027852 |
| 10 | Nenxuan12 | Heilongjiang | Institute of nenjiang, Heilongjiang academy of agriculture | 1970s | 00020564 |
| 11 | Nenxuan14 | Heilongjiang | Institute of nenjiang, Heilongjiang academy of agriculture | 1980s | 00020566 |
| 12 | Nenxuan5 | Heilongjiang | Institute of nenjiang, Heilongjiang academy of agriculture | 1960s | 00000323 |
| 13 | Nenxuan7 | Heilongjiang | Institute of nenjiang, Heilongjiang academy of agriculture | 1970s | 00000324 |
| 14 | Nenxuan6 | Heilongjiang | Institute of nenjiang, Heilongjiang academy of agriculture | 1960s | 00000335 |
| 15 | Shengu1 | Neimenggu | Neimenggu | 2000s | 00027653 |
| 16 | NaihanTG118 | Neimenggu | Neimenggu | 2000s | 00027654 |
| 17 | Huangyu2 | Neimenggu | Neimenggu | 1950s | 00003167 |
| 18 | Keyu1 | Heilongjiang | Keshan institute of agriculture | 1960s | 00000143 |
| 19 | Huangshazi3 | Heilongjiang | Keshan institute of agriculture | 1950s | 00000235 |
| 20 | Jiguang1 | Heilongjiang | Keshan institute of agriculture | 1950s | 00000239 |
| 21 | Jiguang2 | Heilongjiang | Keshan institute of agriculture | 1950s | 00000240 |
| 22 | Kexi23 | Heilongjiang | Keshan institute of agriculture | 1970s | 00000241 |
| 23 | Hangu1 | Heilongjiang | Keshan institute of agriculture | 1960s | 00000317 |
| 24 | Suigu2 | Heilongjiang | Suihua institute of agriculture | 1970s | 00000467 |
| 25 | Suigu1 | Heilongjiang | Suihua institute of agriculture | 1970s | 00000468 |
| 26 | Heguang1 | Heilongjiang | Heguang institute of agriculture | 1950s | 00000488 |
| 27 | Heguang2 | Heilongjiang | Heguang institute of agriculture | 1950s | 00000489 |
| 28 | Heguang3 | Heilongjiang | Heguang institute of agriculture | 1960s | 00000490 |
| 29 | Heguang4 | Heilongjiang | Heguang institute of agriculture | 1960s | 00000491 |
| 30 | Heguang5 | Heilongjiang | Heguang institute of agriculture | 1960s | 00000492 |
| 31 | Heguang6 | Heilongjiang | Heguang institute of agriculture | 1970s | 00000493 |
| 32 | Heguang7 | Heilongjiang | Heguang institute of agriculture | 1970s | 00000494 |
| 33 | Heguang8 | Heilongjiang | Heguang institute of agriculture | 1970s | 00000495 |
| 34 | Xindalihuang1 | Heilongjiang | Mudanjiang institute of agriculture | 1960s | 00000534 |
| 35 | Jingu5 | Liaoning | Jinzhou institute of agriculture | 1970s | 00002772 |
| 36 | Jinfen15 | Shanxi | Institute of economic crops, Shanxi academy of agriculture | 1970s | 00006335 |
| 37 | Zhuxi2 | Hebei | Zhangjiakou institute of agriculture | 1970s | 00007002 |
| 38 | Zhuxi8 | Hebei | Zhangjiakou institute of agriculture | 1970s | 00007081 |
| 39 | Zhuxi12 | Hebei | Zhangjiakou institute of agriculture | 1970s | 00007141 |
| 40 | Zhangnong1 | Hebei | Zhangjiakou institute of agriculture | 1950s | 00007612 |
| 41 | Zhangnong2 | Hebei | Zhangjiakou institute of agriculture | 1950s | 00007613 |
| 42 | Zhangnong3 | Hebei | Zhangjiakou institute of agriculture | 1950s | 00007614 |
| 43 | Zhangnong5 | Hebei | Zhangjiakou institute of agriculture | 1950s | 00007616 |
| 44 | Zhangnong6 | Hebei | Zhangjiakou institute of agriculture | 1950s | 00007617 |
| 45 | Zhangnong7 | Hebei | Zhangjiakou institute of agriculture | 1950s | 00007618 |
| 46 | Zhangnong8 | Hebei | Zhangjiakou institute of agriculture | 1950s | 00007619 |
| 47 | Zhangnong10 | Hebei | Zhangjiakou institute of agriculture | 1960s | 00007621 |
| 48 | Zhangnong11 | Hebei | Zhangjiakou institute of agriculture | 1960s | 00007622 |
| **G1-2** |  |  |  |  |  |
| 1 | Chaogu13 | Liaoning | Institute of water protection, Liaoning academy of agriculture | 2000s | 00027873 |
| 2 | Jingu2 | Liaoning | Jinzhou institute of agriculture | 1960s | 00002770 |
| 3 | Tiegu7 | Liaoning | Tieling institute of agriculture | 1990s | 00026855 |
| 4 | Yusan | Shanxi | Institute of economic crops, Shanxi academy of agriculture | 2000s | 00028001 |
| 5 | Jinfen5 | Shanxi | Institute of economic crops, Shanxi academy of agriculture | 1960s | 00006345 |
| 6 | Jinfen16 | Shanxi | Institute of economic crops, Shanxi academy of agriculture | 1960s | 00006394 |
| 7 | Huanggu2 | Shanxi | Shanxi | 1950s | 00015747 |
| 8 | Jingu8 | Shanxi | Institute of crop breeding, Shanxi academy of agriculture | 1970s | 00027200 |
| 9 | Mengheigu8 | Neimenggu | Neimenggu academy of agriculture | 2000s | 00027883 |
| 10 | Zhaogu6 | Neimenggu | Chifeng institute of agriculture | 1960s | 00002378 |
| 11 | Zhaogu40 | Neimenggu | Chifeng institute of agriculture | 1970s | 00002386 |
| 12 | Zhaogu21 | Neimenggu | Chifeng institute of agriculture | 1970s | 00002387 |
| 13 | Zhaoza11 | Neimenggu | Chifeng institute of agriculture | 1970s | 00002388 |
| 14 | Zhaoza10 | Neimenggu | Chifeng institute of agriculture | 1970s | 00002389 |
| 15 | Zhaonong19 | Neimenggu | Chifeng institute of agriculture | 1970s | 00002392 |
| 16 | Zhaonong33 | Neimenggu | Chifeng institute of agriculture | 1970s | 00002395 |
| 17 | Zhaonong41 | Neimenggu | Chifeng institute of agriculture | 1970s | 00002396 |
| 18 | Zhaonong1 | Neimenggu | Chifeng institute of agriculture | 1970s | 00002397 |
| 19 | Neigu2 | Neimenggu | Institute of crops, Neimenggu academy of agriculture | 1960s | 00015600 |
| 20 | Zhegu8 | Neimenggu | Tongliao institute of agriculture | 1960s | 00015605 |
| 21 | Chigu7 | Neimenggu | Chifeng institute of agriculture | 1980s | 00021304 |
| 22 | Yanxiagu1 | Shannxi | Yan’an institute of agriculture | 1980s | 00004048 |
| 23 | Yangu3 | Shannxi | Yan’an institute of agriculture | 1970s | 00004105 |
| 24 | Yangu2 | Shannxi | Yan’an institute of agriculture | 1970s | 00004122 |
| 25 | Yangu10 | Shannxi | Yan’an institute of agriculture | 1990s | 00021125 |
| 26 | Zhangnong4 | Hebei | Zhangjiakou institute of agriculture | 1950s | 00007615 |
| 27 | Zhangnong9 | Hebei | Zhangjiakou institute of agriculture | 1950s | 00007620 |
| 28 | Shinong2 | Hebei | Institute of millet, Hebei academy of agriculture | 1950s | 00022044 |
| 29 | Shinong3 | Hebei | Institute of millet, Hebei academy of agriculture | 1950s | 00022045 |
| 30 | Huanong1 | Hebei | Institute of millet, Hebei academy of agriculture | 1950s | 00022047 |
| 31 | Bagu1 | Hebei | Zhangjiakou institute of agriculture | 1980s | 00022103 |
| 32 | Bagu2 | Hebei | Zhangjiakou institute of agriculture | 1980s | 00022104 |
| 33 | Bagu5 | Hebei | Zhangjiakou institute of agriculture | 1980s | 00022105 |
| 34 | Bagu6 | Hebei | Zhangjiakou institute of agriculture | 1980s | 00022106 |
| 35 | Yuejin4 | Hebei | Chengde institute of agriculture | 1960s | 00015648 |
| 36 | Ange3 | Henan | Anyang institute of agriculture | 1960s | 00009847 |
| 37 | Ange4 | Henan | Anyang institute of agriculture | 1960s | 00009848 |
| 38 | Heigu1 | Heilongjiang | Heihe institute of agriculture | 1970s | 00000007 |
| 39 | Longxuan1 | Heilongjiang | Institute of nenjiang, Heilongjiang academy of agriculture | 1960s | 00000321 |
| 40 | Heigu2 | Heilongjiang | Heihe institute of agriculture | 1980s | 00015424 |
| 41 | Nenxuan2 | Heilongjiang | Institute of nenjiang, Heilongjiang academy of agriculture | 1960s | 00015434 |
| 42 | Nenxuan4 | Heilongjiang | Institute of nenjiang, Heilongjiang academy of agriculture | 1960s | 00015435 |
| 43 | Nenxuan9 | Heilongjiang | Institute of nenjiang, Heilongjiang academy of agriculture | 1970s | 00015436 |
| 44 | Nenxuan10 | Heilongjiang | Institute of nenjiang, Heilongjiang academy of agriculture | 1970s | 00015437 |
| 45 | Nenxuan11 | Heilongjiang | Institute of nenjiang, Heilongjiang academy of agriculture | 1970s | 00015438 |
| 46 | Nenxuan13 | Heilongjiang | Institute of nenjiang, Heilongjiang academy of agriculture | 1980s | 00020565 |
| 47 | Suigu4 | Heilongjiang | Institute of suihua, Heilongjiang academy of agriculture | 1980s | 00020573 |
| 48 | Longgu26 | Heilongjiang | Institute of breeding, Heilongjiang academy of agriculture | 1980s | 00020590 |
| 49 | Muyu6 | Heilongjiang | Institute of Mudanjiang, Heilongjiang academy of agriculture | 1980s | 00020609 |
| 50 | Lujin5 | Shandong | Institute of crops, Shandong academy of agriculture | 1970s | 00019594 |
| 51 | Lugu3 | Shandong | Institute of crops, Shandong academy of agriculture | 1970s | 00019597 |
| 52 | Lugu4 | Shandong | Institute of crops, Shandong academy of agriculture | 1980s | 00019598 |
| 53 | Jiugu7 | Jilin | Jilin institute of agriculture | 1980s | 00020776 |
| 54 | Gonggu6 | Jilin | Institute of breeding, Jilin academy of agriculture | 1970s | 00020960 |
| 55 | Gonggu7 | Jilin | Institute of breeding, Jilin academy of agriculture | 1970s | 00020961 |
| 56 | Gonggu11 | Jilin | Institute of breeding, Jilin academy of agriculture | 1970s | 00020964 |
| 57 | Gonggu23 | Jilin | Institute of breeding, Jilin academy of agriculture | 1970s | 00020965 |
| 58 | Sigu1 | Jilin | Siping institute of agriculture | 1970s | 00021121 |
| 59 | Sigu3 | Jilin | Siping institute of agriculture | 1980s | 00021123 |
| 60 | Yangu10 | Jilin | Yanbian institute of agriculture | 1980s | 00021125 |
| 61 | Zhongpin6 | Beijing | Chinese academy of agriculture sciences | 1980s | 00022264 |
| 62 | Pinzi1 | Beijing | Chinese academy of agriculture sciences | 1980s | 00022265 |
| 63 | Yangu1 | Beijing | Beijing academy of agriculture | 1970s | 00004146 |
| 64 | Yangu3 | Beijing | Beijing academy of agriculture | 1970s | 00004105 |
| **G1-3** |  |  |  |  |  |
| 1 | Chigu5 | Neimenggu | Neimenggu academy of agriculture | 1980s | 00015593 |
| 2 | Chigu10 | Neimenggu | Neimenggu academy of agriculture | 2000s | 00026866 |
| 3 | Chigu4 | Neimenggu | Neimenggu academy of agriculture | 1980s | 00015592 |
| 4 | Chigu6 | Neimenggu | Neimenggu academy of agriculture | 1990s | 00021303 |
| 5 | Chigu8 | Neimenggu | Neimenggu academy of agriculture | 1990s | 00021336 |
| 6 | Chigu9 | Neimenggu | Neimenggu academy of agriculture | 1980s | 00021340 |
| 7 | Fenggu12 | Neimenggu | Neimenggu academy of agriculture | 2000s | 00027876 |
| 8 | Shanxihonggu | Neimenggu | Neimenggu academy of agriculture | 1980s | 00027878 |
| 9 | Mengfenggu7 | Neimenggu | Institute of crops, Neimenggu academy of agriculture | 2000s | 00027884 |
| 10 | Menghangu9 | Neimenggu | Institute of crops, Neimenggu academy of agriculture | 2000s | 00027882 |
| 11 | Menggu12 | Neimenggu | Institute of crops, Neimenggu academy of agriculture | 2000s | 00027881 |
| 12 | Neigu4 | Neimenggu | Institute of crops, Neimenggu academy of agriculture | 1990s | 00027879 |
| 13 | Zhaonong1 | Neimenggu | Chifeng institute of agriculture | 1950s | 00002375 |
| 14 | Zhaonong2 | Neimenggu | Chifeng institute of agriculture | 1960s | 00002376 |
| 15 | Zhaonong4 | Neimenggu | Chifeng institute of agriculture | 1960s | 00002377 |
| 16 | Zhaonong13 | Neimenggu | Chifeng institute of agriculture | 1970s | 00002380 |
| 17 | Zhaonong10 | Neimenggu | Chifeng institute of agriculture | 1970s | 00002383 |
| 18 | Zhaonong11 | Neimenggu | Chifeng institute of agriculture | 1970s | 00002384 |
| 19 | Zhaoza5 | Neimenggu | Chifeng institute of agriculture | 1970s | 00002385 |
| 20 | Zhaoza9 | Neimenggu | Chifeng institute of agriculture | 1970s | 00002390 |
| 21 | Zhaoza7 | Neimenggu | Chifeng institute of agriculture | 1970s | 00002391 |
| 22 | Zhaonong30 | Neimenggu | Chifeng institute of agriculture | 1970s | 00002393 |
| 23 | Zhaonong15 | Neimenggu | Chifeng institute of agriculture | 1970s | 00002394 |
| 24 | Longgu11 | Gansu | Institute of crops, Gansu academy of agriculture | 2000s | 00028014 |
| 25 | Longgu5 | Gansu | Institute of crops, Gansu academy of agriculture | 1970s | 00018781 |
| 26 | Longgu8 | Gansu | Institute of crops, Gansu academy of agriculture | 1990s | 00025560 |
| 27 | Longgu10 | Gansu | Institute of crops, Gansu academy of agriculture | 1990s | 00028013 |
| 28 | Longgu3 | Gansu | Institute of crops, Gansu academy of agriculture | 1970s | 00028015 |
| 29 | Longgu4 | Gansu | Institute of crops, Gansu academy of agriculture | 1970s | 00018719 |
| 30 | Longgu6 | Gansu | Institute of crops, Gansu academy of agriculture | 1980s | 00025601 |
| 31 | Longgu7 | Gansu | Institute of crops, Gansu academy of agriculture | 1990s | 00028016 |
| 32 | Longgu9 | Gansu | Institute of crops, Gansu academy of agriculture | 1990s | 00025592 |
| 33 | Longsu2 | Gansu | Institute of crops, Gansu academy of agriculture | 1970s | 00003730 |
| 34 | Ange1 | Henan | An’yang institute of agriculture | 1960s | 00009845 |
| 35 | Jingu33 | Shanxi | Institute of high latitude crops, Shanxi academy of agriculture | 2000s | 00027967 |
| 36 | Jingu23 | Shanxi | Institute of high latitude crops, Shanxi academy of agriculture | 1990s | 00027192 |
| 37 | Jingu25 | Shanxi | Institute of high latitude crops, Shanxi academy of agriculture | 1990s | 00027193 |
| 38 | Jingu31 | Shanxi | Institute of high latitude crops, Shanxi academy of agriculture | 2000s | 00027968 |
| 39 | Datong27 | Shanxi | Institute of high latitude crops, Shanxi academy of agriculture | 2000s | 00027969 |
| 40 | Jingu39 | Shanxi | Institute of high latitude crops, Shanxi academy of agriculture | 2000s | 00027970 |
| 41 | Jingu13 | Shanxi | Institute of millet, Shanxi academy of agriculture | 1980s | 00027980 |
| 42 | Jingu11 | Shanxi | Institute of economic crops, Shanxi academy of agriculture | 1970s | 00027998 |
| 43 | Jingu18 | Shanxi | Institute of economic crops, Shanxi academy of agriculture | 1990s | 00024666 |
| 44 | Jinfen6 | Shanxi | Institute of economic crops, Shanxi academy of agriculture | 1970s | 00006363 |
| 45 | Jinfen9 | Shanxi | Institute of economic crops, Shanxi academy of agriculture | 1960s | 00006413 |
| 46 | Jinfen3 | Shanxi | Institute of economic crops, Shanxi academy of agriculture | 1960s | 00006415 |
| 47 | 8322-14 | Hebei | Huailai | 2000s | 00027931 |
| 48 | Heiguzi | Hebei | Chengde institute of agriculture | 1950s | 00027896 |
| 49 | Hongguzi | Hebei | Chengde institute of agriculture | 1950s | 00027897 |
| 50 | Bagu214 | Hebei | Zhangjiakou institute of agriculture | 1990s | 00026938 |
| 51 | Zhuxi13 | Hebei | Zhangjiakou institute of agriculture | 1970s | 00006957 |
| 52 | Zhuxi1 | Hebei | Zhangjiakou institute of agriculture | 1970s | 00007001 |
| 53 | Zhangnong12 | Hebei | Zhangjiakou institute of agriculture | 1960s | 00007623 |
| 54 | Zhangnong13 | Hebei | Zhangjiakou institute of agriculture | 1960s | 00007624 |
| 55 | Zhangnong14 | Hebei | Zhangjiakou institute of agriculture | 1960s | 00007625 |
| 56 | Zhangnong15 | Hebei | Zhangjiakou institute of agriculture | 1970s | 00007626 |
| 57 | Chengnong2 | Hebei | Chengde institute of agriculture | 1950s | 00008085 |
| 58 | Chenggu3 | Hebei | Chengde institute of agriculture | 1970s | 00008059 |
| 59 | Chenggu5 | Hebei | Chengde institute of agriculture | 1970s | 00008060 |
| 60 | Chenggu4 | Hebei | Chengde institute of agriculture | 1970s | 00008061 |
| 61 | Chenggu6 | Hebei | Chengde institute of agriculture | 1970s | 00008062 |
| 62 | Jigu8 | Hebei | Zhangjiakou institute of agriculture | 1980s | 00019165 |
| 63 | Shinong4 | Hebei | Institute of millet, Hebei academy of agriculture | 1950s | 00022046 |
| 64 | Honggaigu | Beijing | Zhongpinkaiyuan | 1990s | 00027846 |
| 65 | Longgu27 | Heilongjiang | Institute of breeding, Heilongjiang academy of agriculture | 1980s | 00020592 |
| 66 | Nenxuan11 | Heilongjiang | Institute of Nenjiang, Heilongjiang academy of agriculture | 1970s | 00015438 |
| 67 | Keyu12 | Heilongjiang | Keshan institute of agriculture | 1970s | 00000145 |
| 68 | Keyu18 | Heilongjiang | Keshan institute of agriculture | 1970s | 00000146 |
| 69 | Kexi30 | Heilongjiang | Keshan institute of agriculture | 1970s | 00000147 |
| 70 | Huangshazi1 | Heilongjiang | Keshan institute of agriculture | 1950s | 00000234 |
| 71 | Xindalihuang2 | Heilongjiang | Mudanjiang institute of agriculture | 1960s | 00000535 |
| 72 | Suigu3 | Heilongjiang | Suihua institute of agriculture | 1980s | 00020572 |
| 73 | Yangu12 | Shannxi | Yan’an institute of agriculture | 1990s | 00028012 |
| 74 | Qingzhenzhu | Shannxi | Yan’an seed station | 2000s | 00028000 |
| 75 | Yangu13 | Shannxi | Yan’an institute of agriculture | 2000s | 461 |
| 76 | Lugu2 | Shandong | Institute of crops, Shandong academy of agriculture | 1970s | 00019586 |
| 77 | Lugu1 | Shandong | Institute of crops, Shandong academy of agriculture | 1960s | 00019591 |
| 78 | Lujin1 | Shandong | Institute of crops, Shandong academy of agriculture | 1970s | 00019592 |
| 79 | Gonggu9 | Jilin | Institute of breeding, Jilin academy of agriculture | 1970s | 00020963 |
| 80 | Gonggu36 | Jilin | Institute of breeding, Jilin academy of agriculture | 1970s | 00020968 |
| 81 | Sigu2 | Jilin | Siping institute of agriculture | 1980s | 00021122 |
| 82 | Yangu9 | Jilin | Yanbian institute of agriculture | 1980s | 00021124 |
| 83 | Yangu2 | Beijing | Beijing academy of agriculture | 1970s | 00022267 |
| 84 | Tiegu8 | Liaoning | Tieling institute of agriculture | 1990s | 00026856 |
| 85 | Ange2 | Henan | An’yang institute of agriculture | 1960s | 00009846 |
| 86 | Ange5 | Henan | An’yang institute of agriculture | 1960s | 00009849 |
| **G2-1** |  |  |  |  |  |
| 1 | Chaogu12 | Liaoning | Institute of water protection, Liaoning academy of agriculture | 2000s | 00027800 |
| 2 | Huangjinmiao | Neimenggu | Chifeng institute of agriculture | 2000s | 00027877 |
| 3 | Mengfenggu11 | Neimenggu | Institute of crops, Neimenggu academy of agriculture | 2000s | 470 |
| 4 | Huangjingu | Hebei | Chengde institute of agriculture | 1950s | 00027893 |
| 5 | Chang0301 | Shanxi | Institute of millet, Shanxi academy of agriculture | 2000s | 00027973 |
| 6 | Changnong35 | Shanxi | Institute of millet, Shanxi academy of agriculture | 2000s | 00027974 |
| 7 | Changnong36 | Shanxi | Institute of millet, Shanxi academy of agriculture | 2000s | 00027975 |
| 8 | Changnong38 | Shanxi | Institute of millet, Shanxi academy of agriculture | 2000s | 00027976 |
| 9 | Changnong39 | Shanxi | Institute of millet, Shanxi academy of agriculture | 2000s | 00027977 |
| 10 | Changsheng06 | Shanxi | Institute of millet, Shanxi academy of agriculture | 2000s | 00027978 |
| 11 | Changsheng07 | Shanxi | Institute of millet, Shanxi academy of agriculture | 2000s | 00027979 |
| 12 | Jingu14 | Shanxi | Institute of millet, Shanxi academy of agriculture | 1980s | 00027981 |
| 13 | Jingu16 | Shanxi | Institute of millet, Shanxi academy of agriculture | 1980s | 00027982 |
| 14 | Jingu20 | Shanxi | Institute of millet, Shanxi academy of agriculture | 1990s | 00024664 |
| 15 | Jingu21 | Shanxi | Institute of millet, Shanxi academy of agriculture | 1990s | 00024668 |
| 16 | Jingu22 | Shanxi | Institute of millet, Shanxi academy of agriculture | 1990s | 00027983 |
| 17 | Jingu27 | Shanxi | Institute of millet, Shanxi academy of agriculture | 1990s | 00027984 |
| 18 | Chang0302 | Shanxi | Institute of millet, Shanxi academy of agriculture | 2000s | 00027985 |
| 19 | Changza2 | Shanxi | Institute of millet, Shanxi academy of agriculture | 2000s | 00027986 |
| 20 | Changnong40 | Shanxi | Institute of millet, Shanxi academy of agriculture | 2011s | 00027987 |
| 21 | Changsheng04 | Shanxi | Institute of millet, Shanxi academy of agriculture | 2000s | 00027988 |
| 22 | Changsheng08 | Shanxi | Institute of millet, Shanxi academy of agriculture | 2000s | 00027989 |
| 23 | Changsheng18 | Shanxi | Institute of millet, Shanxi academy of agriculture | 2000s | 00027990 |
| 24 | Changgu2 | Shanxi | Institute of millet, Shanxi academy of agriculture | 2000s | 00027991 |
| 25 | Changgu4 | Shanxi | Institute of millet, Shanxi academy of agriculture | 2000s | 00027992 |
| 26 | Jingu30 | Shanxi | Institute of millet, Shanxi academy of agriculture | 2000s | 00027817 |
| 27 | Fenxuan8 | Shanxi | Institute of economic crops, Shanxi academy of agriculture | 2000s | 00027994 |
| 28 | Jingu6 | Shanxi | Institute of economic crops, Shanxi academy of agriculture | 1970s | 00006378 |
| 29 | Jingu10 | Shanxi | Institute of economic crops, Shanxi academy of agriculture | 1970s | 00027996 |
| 30 | Jingu20 | Shanxi | Institute of economic crops, Shanxi academy of agriculture | 1990s | 00024667 |
| 31 | Jingu21 | Shanxi | Institute of economic crops, Shanxi academy of agriculture | 1990s | 00024668 |
| 32 | Jingu24 | Shanxi | Institute of economic crops, Shanxi academy of agriculture | 1990s | 00027997 |
| 33 | Jingu26 | Shanxi | Institute of economic crops, Shanxi academy of agriculture | 1990s | 00027815 |
| 34 | Jingu40 | Shanxi | Institute of economic crops, Shanxi academy of agriculture | 1990s | 00027999 |
| 35 | 87-151 | Shanxi | Institute of economic crops, Shanxi academy of agriculture | 1990s | 00028002 |
| 36 | Yangu11 | Shannxi | Yan’an institute of agriculture | 1990s | 00028011 |
| **G2-2** |  |  |  |  |  |
| 1 | Gonggu65 | Jilin | Institute of breeding, Jilin academy of agriculture | 1990s | 00027697 |
| 2 | Gonggu5 | Jilin | Institute of breeding, Jilin academy of agriculture | 1960s | 00020959 |
| 3 | Gonggu31 | Jilin | Institute of breeding, Jilin academy of agriculture | 1970s | 00020967 |
| 4 | Gonggu60 | Jilin | Institute of breeding, Jilin academy of agriculture | 1980s | 00020969 |
| 5 | Gonggu60 | Jilin | Institute of breeding, Jilin academy of agriculture | 1980s | 00020969 |
| 6 | Gonggu71 | Jilin | Institute of breeding, Jilin academy of agriculture | 2000s | 00027701 |
| 7 | Baigu6 | Jilin | Baicheng institute of agriculture | 1990s | 00021146 |
| 8 | Jiugu8 | Jilin | Jilin institute of agriculture | 1990s | 00020777 |
| 9 | Jiugu11 | Jilin | Jilin institute of agriculture | 2000s | 00027867 |
| 10 | Gonggu68 | Jilin | Institute of breeding, Jilin academy of agriculture | 2000s | 00027698 |
| 11 | Gonggu5-1 | Jilin | Institute of breeding, Jilin academy of agriculture | 1970s | 00020972 |
| 12 | Gongguxin7 | Jilin | Institute of breeding, Jilin academy of agriculture | 1970s | 00020962 |
| 13 | Gonggu61 | Jilin | Institute of breeding, Jilin academy of agriculture | 1990s | 00020970 |
| 14 | Gonggu62 | Jilin | Institute of breeding, Jilin academy of agriculture | 1990s | 00020971 |
| 15 | Gonggu63 | Jilin | Institute of breeding, Jilin academy of agriculture | 1990s | 00027861 |
| 16 | Gonggu66 | Jilin | Institute of breeding, Jilin academy of agriculture | 1990s | 00027862 |
| 17 | Gonggu69 | Jilin | Institute of breeding, Jilin academy of agriculture | 2000s | 00027699 |
| 18 | Gonggu70 | Jilin | Institute of breeding, Jilin academy of agriculture | 2000s | 00027700 |
| 19 | Gonggu72 | Jilin | Institute of breeding, Jilin academy of agriculture | 2000s | 00027702 |
| 20 | Gonggu73 | Jilin | Institute of breeding, Jilin academy of agriculture | 2000s | 00027855 |
| 21 | Gonggu74 | Jilin | Institute of breeding, Jilin academy of agriculture | 2000s | 00027856 |
| 22 | Gonggu75 | Jilin | Institute of breeding, Jilin academy of agriculture | 2000s | 00027857 |
| 23 | Gong’Ai4 | Jilin | Institute of breeding, Jilin academy of agriculture | 2000s | 00027858 |
| 24 | Baigu7 | Jilin | Baicheng institute of agriculture | 1990s | 00027853 |
| 25 | Baigu9 | Jilin | Baicheng institute of agriculture | 1990s | 00027854 |
| 26 | Jiugu16 | Jilin | Jilin institute of agriculture | 2000s | 404 |
| 27 | Jiugu18 | Jilin | Jilin institute of agriculture | 2000s | 405 |
| 28 | Jiugu9 | Jilin | Jilin institute of agriculture | 1990s | 00027866 |
| 29 | Jiugu14 | Jilin | Jilin institute of agriculture | 2000s | 00027869 |
| 30 | Jiugu15 | Jilin | Jilin institute of agriculture | 2000s | 00027870 |
| 31 | Jingu35 | Shanxi | Institute of millet, Shanxi academy of agriculture | 2000s | 00027993 |
| 32 | 77-322 | Shanxi | Institute of crops, Shanxi academy of agriculture | 1980s | 00027203 |
| 33 | Jinfen13 | Shanxi | Institute of economic crops, Shanxi academy of agriculture | 1960s | 00006337 |
| 34 | Huangyu3 | Neimenggu | Neimenggu | 1950s | 00002910 |
| 35 | Huangyu4 | Neimenggu | Neimenggu | 1960s | 00003143 |
| 36 | Yangu5 | Shannxi | Yan’an institute of agriculture | 1970s | 00004128 |
| 37 | Yangu1 | Shannxi | Yan’an institute of agriculture | 1960s | 00004146 |
| **G2-3** |  |  |  |  |  |
| 1 | Jigu18 | Hebei | Institute of millet, Hebei academy of agriculture | 2000s | 00027907 |
| 2 | Jigu24 | Hebei | Institute of millet, Hebei academy of agriculture | 2000s | 00027910 |
| 3 | Jigu29 | Hebei | Institute of millet, Hebei academy of agriculture | 2000s | 00027912 |
| 4 | Canggu3 | Hebei | Cangzhou institute of agriculture | 2000s | 00027888 |
| 5 | Canggu4 | Hebei | Cangzhou institute of agriculture | 2000s | 00027889 |
| 6 | Jigu19 | Hebei | Institute of millet, Hebei academy of agriculture | 2000s | 00027908 |
| 7 | Jigu20 | Hebei | Institute of millet, Hebei academy of agriculture | 2000s | 00027695 |
| 8 | Jigu21 | Hebei | Institute of millet, Hebei academy of agriculture | 2000s | 00027696 |
| 9 | Jigu22 | Hebei | Institute of millet, Hebei academy of agriculture | 2000s | 00027909 |
| 10 | Jigu26 | Hebei | Institute of millet, Hebei academy of agriculture | 2000s | 00027911 |
| 11 | Xiaoxiangmi | Hebei | Institute of millet, Hebei academy of agriculture | 2000s | 00027913 |
| 12 | K325 | Hebei | Institute of millet, Hebei academy of agriculture | 2000s | 00027916 |
| 13 | Jigu15 | Hebei | Institute of dry-Land Farming, Hebei academy of agriculture | 1990s | 00027924 |
| 14 | Jigu17 | Hebei | Institute of dry-Land Farming, Hebei academy of agriculture | 1990s | 00027925 |
| 15 | Henggu9 | Hebei | Institute of dry-Land Farming, Hebei academy of agriculture | 2000s | 00027926 |
| 16 | 2015 | Hebei | Institute of dry-Land Farming, Hebei academy of agriculture | 2000s | 00027928 |
| 17 | Shuxiang1 | Hebei | Huailai | 2000s | 00027932 |
| 18 | Cang409 | Hebei | Cangzhou institute of agriculture | 2000s | 00027887 |
| 19 | Cang344 | Hebei | Cangzhou institute of agriculture | 2000s | 291 |
| 20 | Kuan9 | Hebei | Chengde institute of agriculture | 1990s | 00027894 |
| 21 | 03-992 | Hebei | Chengde institute of agriculture | 1990s | 00027895 |
| 22 | Baogu18 | Hebei | Baoding institute of agriculture | 2000s | 00027886 |
| 23 | Jixiang1 | Hebei | Institute of millet, Hebei academy of agriculture | 2000s | 00027914 |
| 24 | Jigu25 | Hebei | Institute of millet, Hebei academy of agriculture | 2000s | 00009273 |
| 25 | Baimi1 | Hebei | Institute of millet, Hebei academy of agriculture | 2000s | 00027915 |
| 26 | Gufeng1 | Hebei | Institute of millet, Hebei academy of agriculture | 1990s | 00027733 |
| 27 | Gufeng2 | Hebei | Institute of millet, Hebei academy of agriculture | 2000s | 00027917 |
| 28 | 95307 | Hebei | Institute of millet, Hebei academy of agriculture | 1990s | 00027918 |
| 29 | Jingu1 | Hebei | Institute of millet, Hebei academy of agriculture | 2000s | 00027919 |
| 30 | Jigu28 | Hebei | Institute of millet, Hebei academy of agriculture | 2000s | 00027921 |
| 31 | Jigu30 | Hebei | Institute of millet, Hebei academy of agriculture | 2000s | 00027922 |
| 32 | 206058 | Hebei | Institute of millet, Hebei academy of agriculture | 2000s | 00027923 |
| 33 | Cang555 | Hebei | Cangzhou institute of agriculture | 2000s | 00027890 |
| 34 | Jigu12 | Hebei | Cangzhou institute of agriculture | 1990s | 00027891 |
| 35 | Cang156 | Hebei | Cangzhou institute of agriculture | 2000s | 361 |
| 36 | Chenggu11 | Hebei | Chengde institute of agriculture | 2000s | 00027898 |
| 37 | Chenggu12 | Hebei | Chengde institute of agriculture | 2000s | 00027899 |
| 38 | Baai2 | Hebei | Zhangjiakou institute of agriculture | 1990s | 00022085 |
| 39 | Badi1 | Hebei | Zhangjiakou institute of agriculture | 1990s | 00026939 |
| 40 | Yugu11 | Henan | An’yang institute of agriculture | 2000s | 00027948 |
| 41 | Yugu7 | Henan | An’yang institute of agriculture | 1990s | 00024190 |
| 42 | Yugu14 | Henan | An’yang institute of agriculture | 2000s | 00027953 |
| 43 | Zheng05-2 | Henan | Institute of crops, Henan academy of agriculture | 2000s | 00027934 |
| 44 | An04-4705 | Henan | An’yang institute of agriculture | 2000s | 00027935 |
| 45 | An04-5014 | Henan | An’yang institute of agriculture | 2000s | 00027937 |
| 46 | An06-4113 | Henan | An’yang institute of agriculture | 2000s | 00027939 |
| 47 | An07-Hai4065 | Henan | An’yang institute of agriculture | 2000s | 00027945 |
| 48 | An06h-8023 | Henan | An’yang institute of agriculture | 2000s | 00027947 |
| 49 | Yugu9 | Henan | An’yang institute of agriculture | 2000s | 00027949 |
| 50 | Yugu1 | Henan | An’yang institute of agriculture | 2000s | 00024169 |
| 51 | Yugu2 | Henan | An’yang institute of agriculture | 1980s | 00024170 |
| 52 | GS-yugu5 | Henan | An’yang institute of agriculture | 1990s | 00024186 |
| 53 | An93-15 | Henan | An’yang institute of agriculture | 2000s | 00027950 |
| 54 | Lugu5 | Shandong | Institute of crops, Shandong academy of agriculture | 1980s | 00019599 |
| 55 | Lugu8 | Shandong | Institute of crops, Shandong academy of agriculture | 1980s | 00019601 |
| 56 | Lugu10 | Shandong | Institute of crops, Shandong academy of agriculture | 1990s | 00027184 |
| 57 | Jigu12 | Shandong | Institute of crops, Shandong academy of agriculture | 2000s | 00027960 |
| 58 | Ji07607 | Shandong | Institute of crops, Shandong academy of agriculture | 2000s | 00027958 |
| 59 | Ji07610 | Shandong | Institute of crops, Shandong academy of agriculture | 2000s | 317 |
| 60 | Lugu6 | Shandong | Institute of crops, Shandong academy of agriculture | 1980s | 00019862 |
| 61 | Lugu7 | Shandong | Institute of crops, Shandong academy of agriculture | 1980s | 00019600 |
| 62 | Lugu9 | Shandong | Institute of crops, Shandong academy of agriculture | 1980s | 00019863 |
| 63 | Jigu11 | Shandong | Institute of crops, Shandong academy of agriculture | 2000s | 00027959 |
| 64 | Jigu13 | Shandong | Institute of crops, Shandong academy of agriculture | 2000s | 00027961 |
| 65 | Jigu14 | Shandong | Institute of crops, Shandong academy of agriculture | 2000s | 00027962 |
| 66 | Lujin3 | Shandong | Institute of crops, Shandong academy of agriculture | 1970s | 00019593 |
| 67 | Gongai2 | Jilin | Institute of crops, Jilin academy of agriculture | 2000s | 00027703 |
| 68 | Gongai3 | Jilin | Institute of crops, Jilin academy of agriculture | 2000s | 00027704 |
| 69 | Baigu6 | Jilin | Baicheng institute of agriculture | 1990s | 00021146 |
| 70 | Pin141 | Beijing | Seed station of Fangshan | 2000s | 329 |
| 71 | Jinggu1 | Beijing | Chinese academy of agriculture sciences | 1960s | 00022262 |
| 72 | Jinsuigu1 | Hebei | Institute of millets, Hebei academy of agriculture | 2000s | 00028017 |
| 73 | Chaogu14 | Liaoning | Institute of water protection, Liaoning academy of agriculture | 2000s | 00027874 |
| 74 | Chaogu15 | Liaoning | Institute of water protection, Liaoning academy of agriculture | 2000s | 00027875 |
| 75 | Tiegu5 | Liaoning | Tieling institute of agriculture | 1980s | 00021152 |
| 76 | Yangu4 | Shannxi | Yan’an institute of agriculture | 1970s | 00004139 |
| 77 | Jinfen2 | Shanxi | Institute of economic crops, Shanxi academy of agriculture | 1960s | 00006414 |
