# Supplementary material for: Microsatellite Variations of Elite Setaria Varieties Released during Last Six Decades in China
Source: PLoS One. 2015 May 1;10(5):e0125688. doi: 10.1371/journal.pone.0125688 (PMC4416935; doi:10.1371/journal.pone.0125688)
Supplement: S2 Table — (DOC) [file pone.0125688.s011.doc]

**Table S2.** Associations of genomic regions under selection (>97.5%) during modern improvement process and agronomic traits detected by GWAS analysis (P<0.05) in foxtail millet.

| **SSR Loci**  **(*Fst* value)** | **Trait** | **E1** | | **E2** | | **E3** | | **E4** | |
| --- | --- | --- | --- | --- | --- | --- | --- | --- | --- |
|  |  | R^2^ | *P* value | R^2^ | *P* value | R^2^ | *P* value | R^2^ | *P* value |
| **B263**  **(0.0641)** | PDL | 0.1294 | 0.0065 | — | — | 0.1151 | 0.0205 | — | — |
| **X298**  **(0.1785)** | PH | — | — | 0.0260 | 0.0384 | 0.0390 | 0.0408 | 0.0054 | 0.0262 |
|  | PD | — | — | — | — | 0.0390 | 0.0030 | 0.1352 | 2.56E-07 |
|  | HD | 0.0334 | 0.0208 | — | — | 0.0294 | 0.0300 | 0.0251 | 0.0045 |
|  | SD | — | — | 0.0349 | 0.0194 | — | — | 0.0387 | 0.0211 |
| **P33**  **(0.1833)** | PH | — | — | 0.0592 | 4.76E-04 | — | — | — | — |
|  | PL | 0.0366 | 0.0116 | 0.0680 | 2.14E-04 | 0.0793 | 0.0012 | — | — |
|  | PDL | — | — | 0.0654 | 0.0043 | — | — | — | — |
|  | PD | — | — | 0.0578 | 0.0240 | 0.0438 | 0.0063 | 0.0682 | 0.0083 |
|  | SD | — | — | — | — | 0.0307 | 0.0120 | — | — |
|  | PW | — | — | — | — | 0.0351 | 0.0226 | — | — |
|  | GW | — | — | 0.0607 | 0.0143 | 0.0302 | 0.0445 | — | — |
| **P58**  **(0.0505)** | PH | — | — | — | — | — | — | 0.0565 | 8.47E-04 |
|  | PL | 0.0734 | 0.0032 | — | — | 0.0901 | 0.0202 | — | — |
|  | HD | 0.0573 | 0.0386 | — | — | — | — | — | — |
|  | PW | — | — | — | — | — | — | 0.0678 | 0.0357 |
| **P61**  **(0.0997)** | PH | — | — | — | — | 0.0955 | 0.0094 | 0.0606 | 0.0032 |
|  | PL | 0.1757 | 1.70E-06 | — | — | — | — | — | — |
|  | PDL | — | — | — | — | 0.0951 | 0.0243 | — | — |
|  | PD | — | — | — | — | 0.0623 | 0.0071 | 0.1094 | 0.0030 |
|  | SD | — | — | — | — | 0.0752 | 9.69E-05 | 0.0750 | 0.0240 |
|  | PW | 0.0634 | 0.0346 | — | — | — | — | — | — |
|  | GW | 0.0837 | 0.0167 | — | — | — | — | — | — |
| **P17x**  **(0.0793)** | PD | 0.0645 | 0.0386 | — | — | — | — | — | — |
|  | SD | — | — | — | — | 0.0284 | 0.0262 | — | — |
| **P4**  **(0.1725)** | PH | — | — | — | — | — | — | 0.0621 | 0.0050 |
|  | HD | — | — | 0.0757 | 0.0476 | — | — | — | — |
|  | PW | 0.1067 | 0.0281 | — | — | — | — | — | — |
|  | GW | 0.1320 | 0.0054 | — | — | — | — | — | — |
| **B126**  **(0.0565)** | PH | — | — | — | — | — | — | 0.0370 | 0.0249 |
|  | PL | — | — | 0.0608 | 0.0167 | 0.0766 | 0.0520 | — | — |
|  | PDL | — | — | 0.0652 | 0.0326 | — | — | — | — |
|  | HD | 0.1196 | 2.43E-04 | — | — | — | — | — | — |
|  | SD | 0.0893 | 0.0479 | — | — | 0.0430 | 0.0115 | — | — |
| **P8**  **(0.0813)** | PH | — | — | — | — | — | — | 0.0496 | 0.0124 |
|  | PL | 0.1413 | 1.41E-05 | 0.1159 | 0.0069 | 0.2409 | 2.63E-08 | 0.1778 | 5.34E-05 |
|  | PDL | 0.0942 | 0.0261 | 0.0442 | 0.0012 | 0.0995 | 0.0094 | — | — |
|  | HD | — | — | 0.0776 | 0.0028 | — | — | — | — |
|  | SD | — | — | — | — | 0.0628 | 0.0055 | — | — |
| **MPGA50**  **(0.1498)** | PH | 0.1133 | 0.0174 | — | — | — | — | — | — |
|  | PD | 0.1259 | 0.0174 | — | — | — | — | — | — |
|  | HD | — | — | 0.0863 | 0.0078 | — | — | 0.0372 | 0.0132 |
|  | GW | 0.0836 | 0.0352 | 0.1047 | 0.0010 | — | — | — | — |
| **MPGA31**  **(0.1799)** | PH | 0.0364 | 0.0164 | 0.0649 | 0.0177 | 0.0906 | 0.0243 | 0.0328 | 0.0289 |
|  | PL | 0.1150 | 0.0020 | — | — | — | — | — | — |
|  | PDL | — | — | — | — | — | — | 0.0696 | 0.0077 |
|  | SD | 0.1972 | 2.60E-04 | — | — | — | — | — | — |
|  | PW | 0.1073 | 0.0032 | — | — | — | — | — | — |
|  | GW | 0.0976 | 0.0065 | — | — | — | — | — | — |
|  |  | | | | | | | | |

Abbreviations:

PH: Plant Height; PL: Panicle Length; PDL: Peduncle Length; PD: Panicle Diameter; HD: Heading Date; SD: Stem Diameter; PW: Panicle Weight; GW: Grain Weight
